# Supplementary material for: AFM imaging reveals the unreconstructed α‑Al2O3(0001) surface to be inhomogeneous and rough
Source: Nat Commun. 2026 May 27;17:4692. doi: 10.1038/s41467-026-73690-0 (PMC13216582; doi:10.1038/s41467-026-73690-0)
Supplement: Supplementary file 1 — Supplementary information [file 41467_2026_73690_MOESM1_ESM.pdf]

**Supplementary information for**

# AFM imaging reveals the unreconstructed α-$Al_2O_3$(0001) surface to be inhomogeneous and rough

Johanna I. Hütner-Reisch,[†,1] Andrea Conti,[†,1] David Kugler,[1] Florian Mittendorfer,[1] Michael Schmid,[1] Ulrike Diebold,[1] Jan Balajka*[,1]

[†] These authors contributed equally to this work.
[1] Institute of Applied Physics, TU Wien, Vienna, Austria
* e-mail: jan.balajka@tuwien.ac.at

This file includes:

## Supplementary figures

- Supplementary Fig. 1 | Ambient AFM images of the $Al_2O_3$(0001) surface before and after annealing in air ........ 2
- Supplementary Fig. 2 | Direct transformation of the unreconstructed $Al_2O_3$(0001) surface into the (√31 × √31)$R$±9° reconstruction ........ 3
- Supplementary Fig. 3 | Survey XPS spectrum of the unreconstructed $Al_2O_3$(0001) surface annealed in vacuum ........ 4
- Supplementary Fig. 4 | Nc-AFM "fingerprint" image of the (2 × 1) oxygen superstructure on Cu(110) used to verify the tip termination ........ 5
- Supplementary Fig. 5 | Height-dependent nc-AFM images of the $Al_2O_3$(0001)-(1 × 1) surface ........ 6
- Supplementary Fig. 6 | DFT-calculated electrostatic potential and vertical electric field near the $Al_2O_3$(0001)-(1 × 1) surface ........ 7
- Supplementary Fig. 7 | XPS spectra of the unreconstructed $Al_2O_3$(0001) surface recorded at different emission angles ........ 8
- Supplementary Fig. 8 | Comparison of grazing-emission XPS spectra of the unreconstructed and (√31 × √31)$R$±9° reconstructed $Al_2O_3$(0001) surfaces ........ 9
- Supplementary Fig. 9 | Island size increases only when the (√31 × √31)$R$±9° reconstruction starts to form ........ 10
- Supplementary Fig. 10 | Stability of the (√31 × √31)$R$±9° reconstructed $Al_2O_3$(0001) surface under oxidizing conditions and water exposure ........ 11
- Supplementary Fig. 11 | LEED images and diffuse background of unreconstructed vs. reconstructed $Al_2O_3$(0001). ........ 12
- Supplementary Fig. 12 | The energetic cost of forming steps on the $Al_2O_3$(0001) surface is low ........ 13
- Supplementary Fig. 13 | Dehydration of an OH-terminated $Al_2O_3$(0001) surface requires mass transport and leads to surface roughening. ........ 14

## Supplementary notes

- Supplementary Note 1 | Additional information for Fig. 2 ........ 15

**Supplementary references** ........ 16

## Supplementary figures

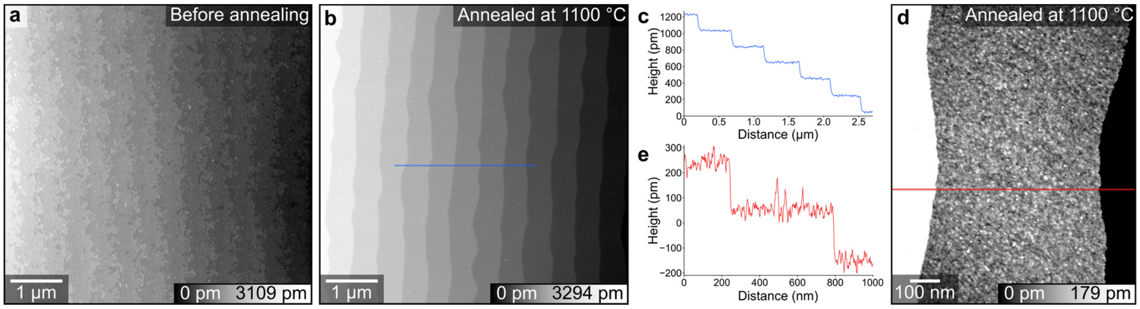


**Supplementary Fig. 1 | Ambient AFM images of the $Al_2O_3$(0001) surface before and after annealing in air.** AFM images were acquired under ambient conditions using a cantilever-based AFM operated in tapping mode. (**a**) Before annealing, the surface exhibits a rough morphology with irregular, corrugated step edges. (**b**) After annealing in a tube furnace at 1100 °C in air for 10 h, smooth step edges and flat terraces are observed. (**c**) The height profile along the blue line in (b), averaged over the line width of five pixels, shows a monoatomic step height of ≈ 220 pm and an average terrace width of ≈ 450 nm. (**d**) Detailed AFM image of the annealed sample, with contrast adjusted to highlight residual roughness within a single terrace. (**e**) Height profile taken along the red line in (d), showing considerable roughness within individual atomic terraces. Due to the finite sharpness of the tip and the short lateral scale of the roughness (cf. Fig. 2a in the main text), the true peak-valley roughness is expected to strongly exceed the height differences in the line scan.

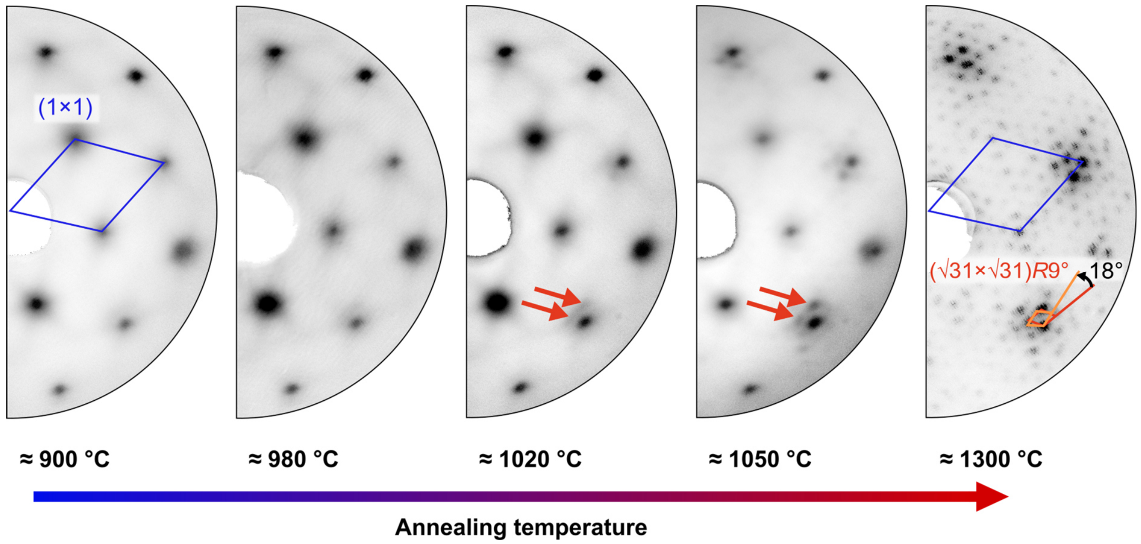


**Supplementary Fig. 2 | Direct transformation of the unreconstructed $Al_2O_3$(0001) surface into the (√31 × √31)*R*±9° reconstruction.** LEED patterns (120 eV) acquired after annealing the unreconstructed surface at progressively higher temperatures from 900 °C (left) to 1300 °C (right), each for approximately 45 min in either $1 \times 10^{-6}$ mbar $O_2$ or UHV (900–1050 °C in $1 \times 10^{-6}$ mbar $O_2$; 1300 °C in UHV). Further experiments showed that the oxygen partial pressure during annealing does not affect the resulting LEED patterns. The onset of the reconstruction is indicated by the emergence of additional diffraction spots (red arrows). The (1 × 1) bulk unit cell is marked in blue; the two (√31 × √31) reconstruction domains, rotated by ±9°, are highlighted in orange and red. The (1 × 1) bulk unit cell (0.475 nm, 120° in real space) is marked in blue.

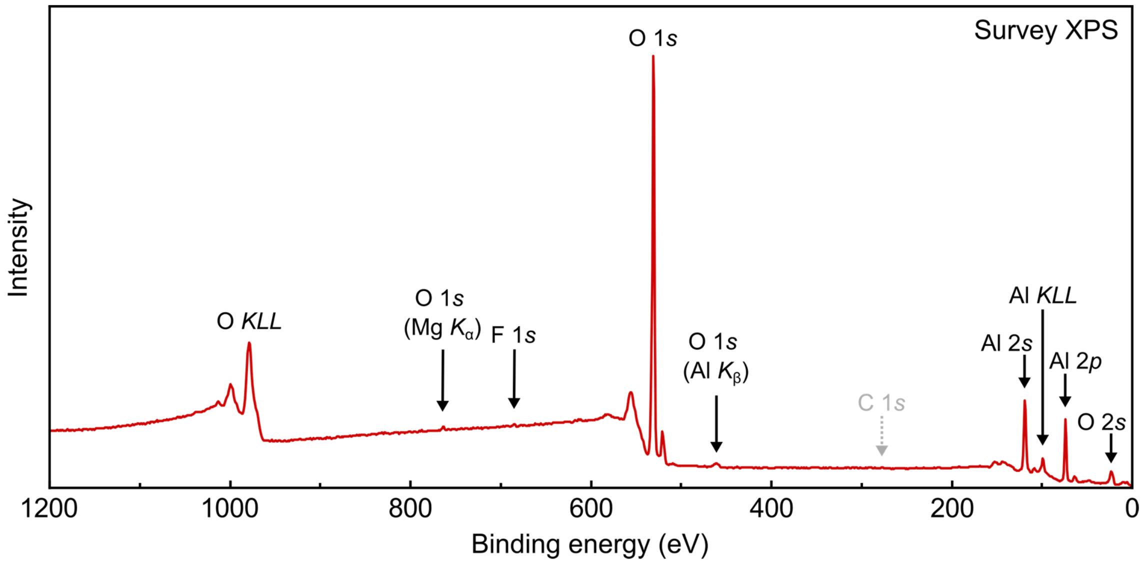


**Supplementary Fig. 3 | Survey XPS spectrum of the unreconstructed $Al_2O_3$(0001) surface annealed in vacuum.** The spectrum was recorded on a sample annealed at ≈ 900 °C (corresponding to the AFM image in Fig. 2d) with a pass energy of 60 eV and an emission angle of 70° from the surface normal. Transitions originating from the $Al_2O_3$ sample are labeled in black. A small F 1*s* peak is present in this spectrum but was not observed on other samples with the same surface structure and is therefore not considered to affect the morphology. The O 1*s* (Mg $K_\alpha$) ghost peak stems from X-rays emitted by the Mg anode adjacent to the Al anode in the dual-anode X-ray source used in this experiment. A spurious C 1*s* signal (indicated in gray) is caused by a measurement artifact and does not originate from the sample, as it remained unchanged with varying sample potential. The spectrum was corrected for surface charging by shifting the energy axis such that the O 1*s* binding energy is 531.0 eV.

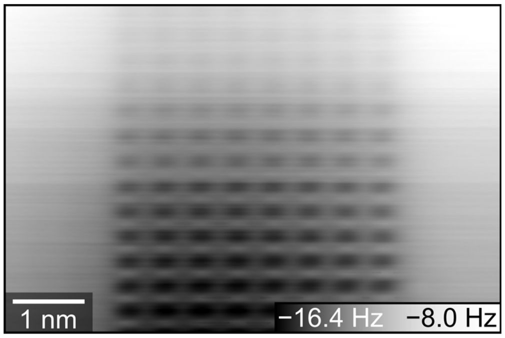


**Supplementary Fig. 4 | Nc-AFM "fingerprint" image of the (2 × 1) oxygen superstructure on Cu(110) used to verify the tip termination.** The image was recorded with the same tip used to image the unreconstructed $Al_2O_3$(0001) surface shown in Fig. 2d in the main text. The fingerprint confirms the CuOx tip termination[1] and supports the assignment of the attractive species on the unreconstructed $Al_2O_3$(0001) surface as Al cations. The image was acquired in constant-height mode at 5 K with an oscillation amplitude of 500 pm and a sample bias of 0 V. The increase in contrast toward the bottom of the image is due to a decreased tip–sample distance.

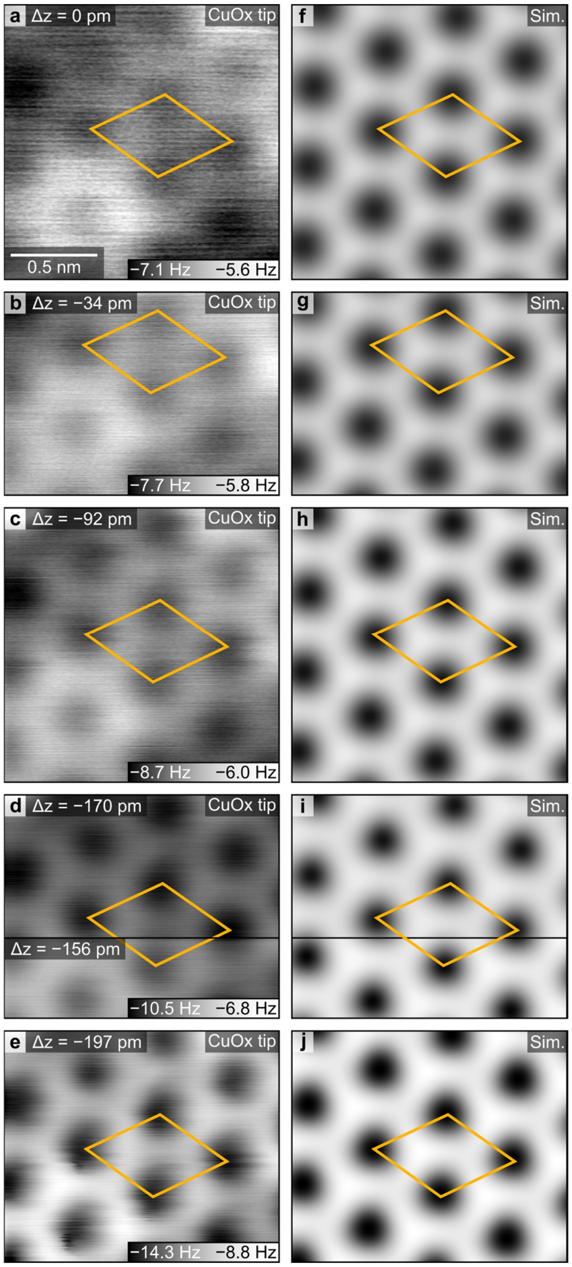


**Supplementary Fig. 5 | Height-dependent nc-AFM images of the $Al_2O_3$(0001)-(1 × 1) surface.** (**a–e**) Experimental constant-height nc-AFM images acquired with a CuOx tip at progressively smaller tip–sample separations; Δ*z* indicates the height difference relative to the first image (a). The contrast remains qualitatively unchanged over the accessible distance range. At closest approach (e), strong tip–surface interactions lead to imaging instabilities, visible as horizontal streaks. Therefore, the Pauli repulsion regime was not reached experimentally, and the images shown in the manuscript were acquired at tip–sample distances dominated by electrostatic forces. (**f–j**) Simulated AFM images calculated for corresponding increments of tip–sample separation (between 700 and 500 pm) reproduce the qualitative contrast.

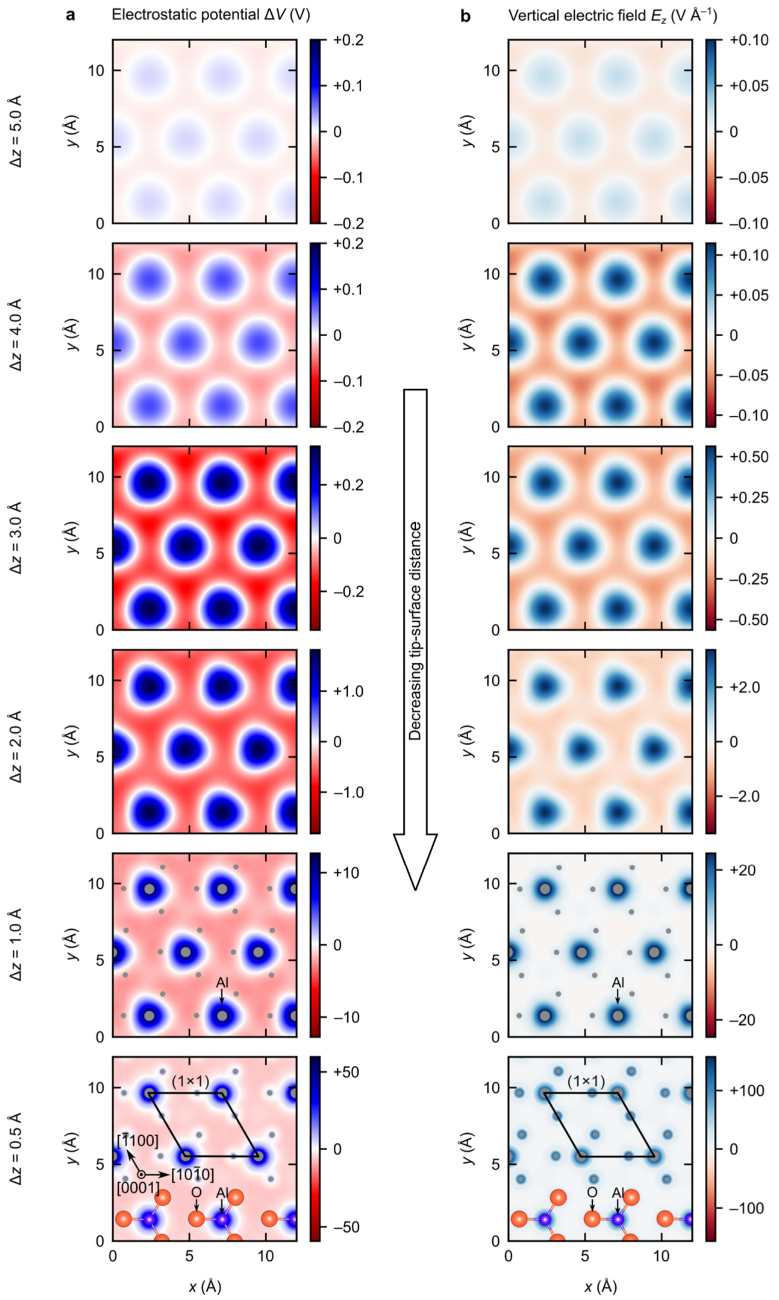


**Supplementary Fig. 6 | DFT-calculated electrostatic potential and vertical electric field near the $Al_2O_3$(0001)-(1 × 1) surface.** (**a**,**b**) Two-dimensional maps (12 Å × 12 Å) of (**a**) the local electrostatic potential $\Delta V$ and (**b**) the vertical electric field component $E_z$, calculated above the surface of the relaxed structure model (Supplementary Data 2) using DFT. Each row corresponds to a vertical distance $\Delta z$ relative to the plane defined by the topmost surface Al atoms, where $\Delta z = z_{\mathrm{slice}} - z_{\mathrm{surf,Al}}$. The electrostatic potential $\Delta V$ was referenced to the vacuum level ($V_{\mathrm{vac}} = 0$) by subtracting the planar-averaged potential evaluated at the center of the vacuum region (≈ 6.87 V above the calculated Fermi level). The vertical electric field $E_z$ was computed as the negative gradient of the electrostatic potential along the surface normal ($E_z = -\partial V/\partial z$), with the positive $z$-direction pointing from the slab into the vacuum. In the bottom two rows ($\Delta z \leq 1.0$ Å), the field in the vicinity of the Al cores cannot be accurately calculated by the PAW approach[2,3]; these regions are indicated by gray disks. The bottom panels ($\Delta z = 0.5$ Å) include an overlay of the relaxed surface atomic structure (Al: blue; O: red), with the (1 × 1) unit cell outlined in black. At tip–surface distances relevant for nc-AFM imaging ($\Delta z \geq 3.0$ Å), the contrast in both $\Delta V$ and $E_z$ is dominated by the surface Al atoms, consistent with the experimentally observed contrast (Figs. 2c,d).

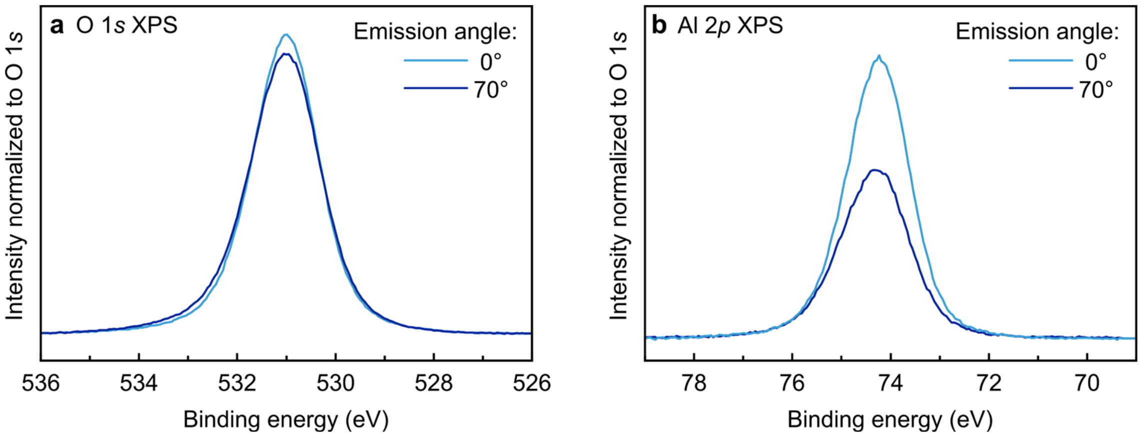


**Supplementary Fig. 7 | XPS spectra of the unreconstructed $Al_2O_3(0001)$ surface recorded at different emission angles.** The spectra were acquired at a pass energy of 20 eV at 0° (normal emission, light blue line) and 70° relative to the surface normal (grazing emission, dark blue line) to increase surface sensitivity. Sample charging was corrected by applying a uniform energy shift to all spectra to align the O 1*s* peak to a binding energy (BE) of 531.0 eV. After background subtraction, each spectrum was normalized to the integrated area of the corresponding O 1*s* peak. The Al 2*p* spectra were divided by the same normalization factor as the O 1*s* to allow comparison of relative Al contributions at different emission angles. (**a**) O 1*s* and (**b**) Al 2*p* spectra of the unreconstructed $Al_2O_3(0001)$ surface after annealing at ≈ 900 °C in $1 \times 10^{-6}$ mbar $O_2$. The absence of an OH-related shoulder at the high-BE side of the O 1*s* peaks shows that the surface is not hydroxylated. Quantitative analysis of the peak areas using atomic sensitivity factors yields [O]/[Al] ratios of 1.52 (0°) and 2.41 (70°). The reduced Al 2*p* intensity at 70° emission indicates a lower relative Al concentration near the surface, consistent with a predominantly oxygen-terminated outermost layer. Photoelectron diffraction effects may additionally contribute to the reduced Al contribution.

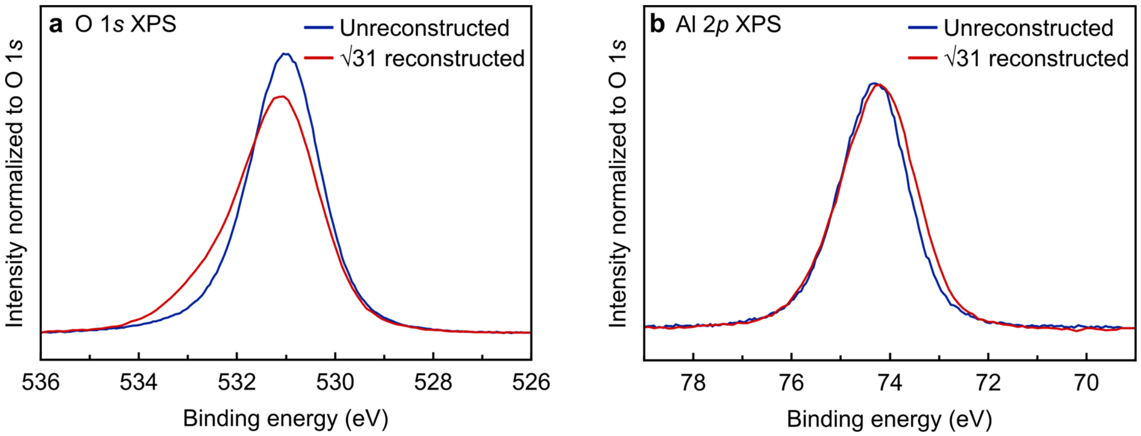


**Supplementary Fig. 8 | Comparison of grazing-emission XPS spectra of the unreconstructed and (√31 × √31)*R*±9° reconstructed $Al_2O_3$(0001) surfaces.** The spectra were acquired at a pass energy of 20 eV at an emission angle of 70° from the surface normal to enhance surface sensitivity. Sample charging was corrected by applying a uniform energy shift to all spectra to align the O 1*s* peak to 531.0 eV. After background subtraction, each spectrum was normalized to the integrated area of the corresponding O 1*s* peak. The Al 2*p* spectra were divided by the same normalization factor to allow comparison of relative Al contributions between the two surfaces. (**a**) O 1*s* and (**b**) Al 2*p* spectra of the unreconstructed $Al_2O_3$(0001) surface (blue, annealed at ≈ 900 °C in $1 \times 10^{-6}$ mbar $O_2$) and the (√31 × √31)*R*±9° reconstructed surface (red, annealed at ≈ 1300 °C in $1 \times 10^{-6}$ mbar $O_2$). Quantitative analysis using atomic sensitivity factors yields [O]/[Al] ratios of 2.41 for the unreconstructed surface and 2.19 for the reconstructed surface (both at 70° emission). The similar relative Al 2*p* intensities indicate that both surfaces are predominantly oxygen-terminated and exhibit comparable stoichiometry.

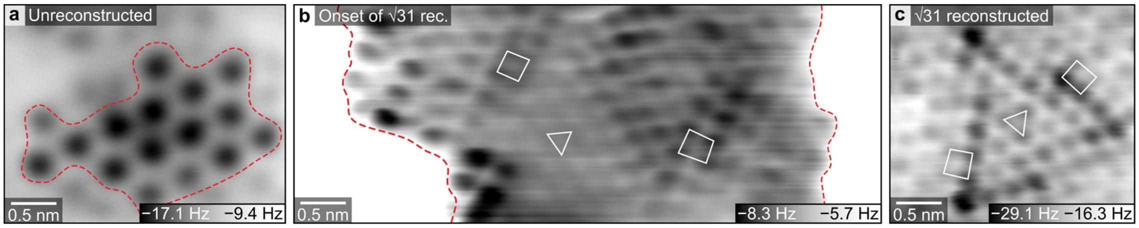


**Supplementary Fig. 9 | Island size increases only when the (√31 × √31)*R*±9° reconstruction starts to form.** (**a**) Nc-AFM image of a representative (1 × 1) island (≈ 2.8 nm wide) on the unreconstructed $Al_2O_3$(0001) surface (see Fig. 2 in the main text). The lateral size of the (1 × 1) islands did not increase with annealing temperature. The image was acquired in constant height with a CuOx-terminated tip; Al atoms appear dark (attractive). (**b**) After prolonged annealing at 820 °C in 1 × $10^{-6}$ mbar $O_2$ for 2.7 h, the islands grew (here ≈ 10 nm wide), but no longer exhibited the (1 × 1) structure. Instead, they displayed structural motifs characteristic of the (√31 × √31)*R*±9° reconstruction[4]: octahedrally coordinated surface Al atoms surrounded by four O atoms in a square geometry (white squares), and tetrahedrally coordinated Al atoms surrounded by three O atoms (white triangles). Island coarsening thus coincides with the onset of the reconstruction. The nc-AFM image was recorded with an oscillation amplitude of 500 pm and 1.5 V sample bias. The contrast is adjusted to highlight features on the island; the lower terrace (white) is not resolved. (**c**) Fully reconstructed (√31 × √31)*R*±9° surface obtained by annealing at 1300 °C in 1 × $10^{-6}$ mbar $O_2$ for 2.5 h, showing an atomically flat morphology and well-ordered structure. At this stage, terrace widths exceeded 100 nm. Images (b) and (c) were acquired in constant height with a Cu-terminated tip; surface O atoms appear dark (attractive).

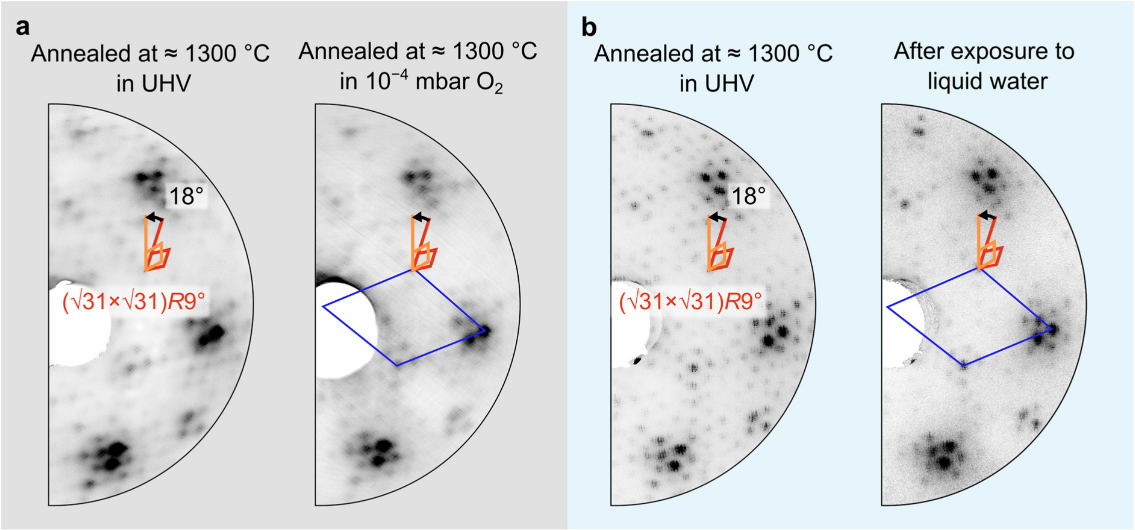


**Supplementary Fig. 10 | Stability of the (√31 × √31)*R*±9° reconstructed $Al_2O_3$(0001) surface under oxidizing conditions and water exposure.** (**a**) LEED patterns (120 eV) acquired before and after annealing in $10^{-4}$ mbar $O_2$, and (**b**) before and after exposure to ultrapure liquid water[5,6], show no change in the reconstruction pattern, demonstrating the irreversibility and environmental stability of the (√31 × √31)*R*±9° surface. Improved focusing of the electron optics yielded enhanced contrast in (b). The (1 × 1) bulk unit cell (0.475 nm, 120° in real space) is marked in blue.

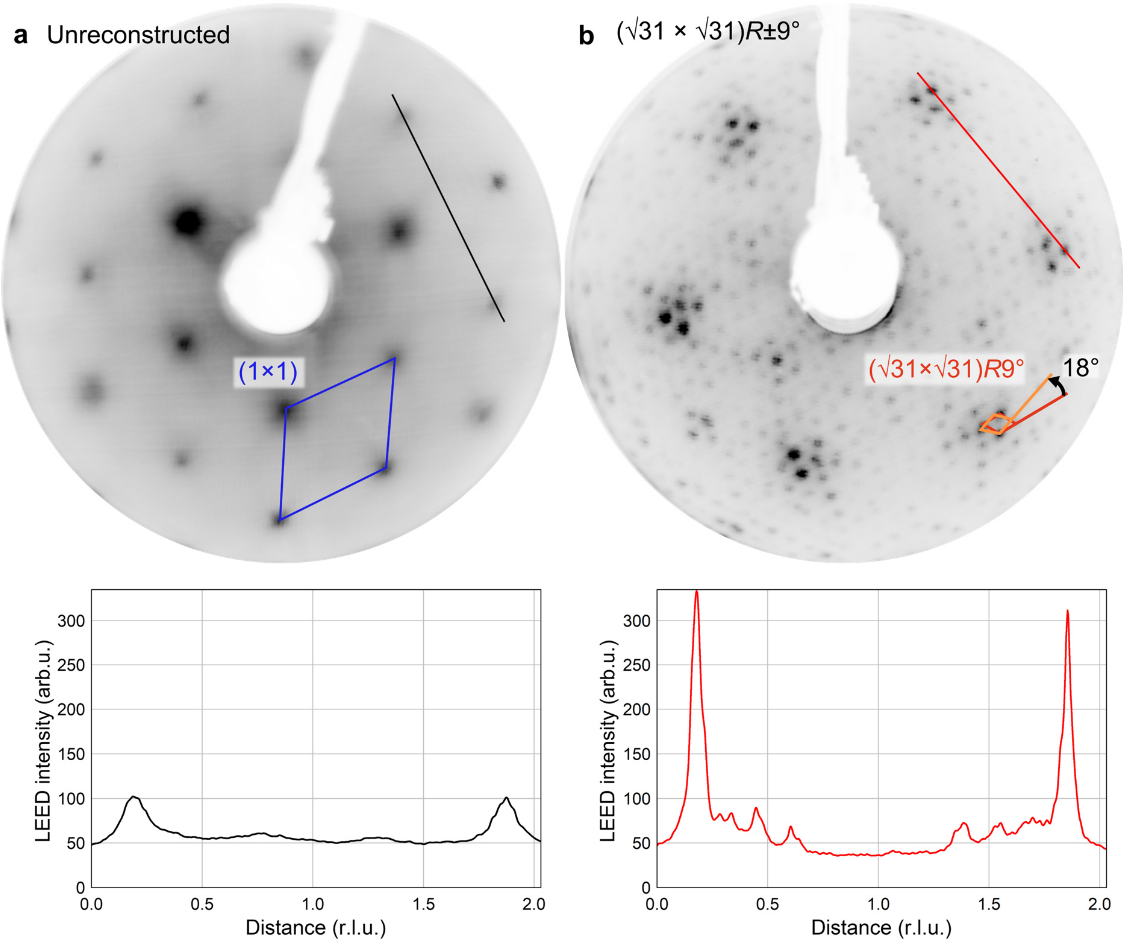


**Supplementary Fig. 11 | LEED images and diffuse background of unreconstructed vs. reconstructed $Al_2O_3$(0001).** LEED images (acquired at room temperature, $E = 120$ eV, inverted grayscale) of (**a**) the unreconstructed $Al_2O_3$(0001) surface annealed at 900 °C and (**b**) the $(\sqrt{31} \times \sqrt{31})R\pm9°$ reconstructed surface annealed at 1300 °C (same data as Supplementary Fig. 2, but without flat-field correction). Some diffraction spots of the unreconstructed surface are broader than others, as expected for a surface with a high density of steps (broad spots occur under out-of-phase conditions[7]). Corresponding intensity profiles along the indicated lines are shown below; distances along the lines are given in reciprocal lattice units (r.l.u.). For the profile plots, the intensities were normalized such that the integrated intensity of all diffraction peaks (after background subtraction) is equal for both images. The unreconstructed surface exhibits broader and less intense diffraction peaks and an enhanced diffuse background, in contrast to the sharp and intense peaks with lower background observed for the reconstructed surface. These results are consistent with the rough and irregular morphology observed by nc-AFM (Fig. 2 in the main text) and supports the interpretation that the observed (1 × 1) pattern primarily reflects the underlying bulk lattice. The (1 × 1) bulk unit cell (0.475 nm, 120° in real space) is marked in blue.

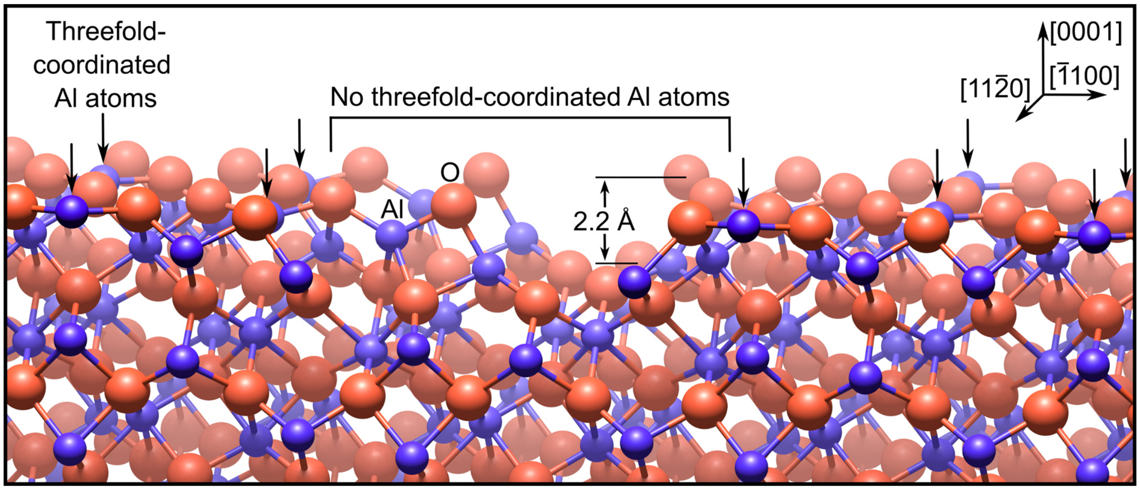


**Supplementary Fig. 12 | The energetic cost of forming steps on the $Al_2O_3$(0001) surface is low.** The lowest-energy structure with steps (Supplementary Data 3) identified in the parallel tempering simulations is higher in energy by 0.93 eV per (2 × 6) simulation cell compared with the relaxed (1 × 1) surface (Fig. 1c in the main text). The (2 × 6) simulation cell contains two steps (one up and one down, each 9.5 Å long). The calculated energy difference therefore corresponds to a step energy of ≈ 49 meV $Å^{-1}$. For comparison, forming a hypothetical, vertical facet of a monoatomic step height (≈ 2.2 Å), using the surface energy of the relaxed (1 × 1) configuration (120 meV $Å^{-2}$, Fig. 1d), would yield a step energy of ≈ 264 meV $Å^{-1}$. Thus, the calculated step energy of ≈ 49 meV $Å^{-1}$ is remarkably low. As only a limited number of configurations were examined, structures with even lower step energies may exist. The atomic configuration helps rationalize the low energy: near the step and on the lower terrace, only oxygen atoms are exposed at the surface, while aluminum atoms are at least fourfold coordinated. The same tendency to avoid threefold-coordinated Al drives the formation of the thermodynamically stable (√31 × √31)*R*±9° reconstruction[4]. The low step energy indicates that the driving force for flattening a rough surface is weak. At the same time, it implies that atoms at the step occupy low-energy (strongly bound) configurations, which reduces step mobility. These considerations may explain why, in our experiments, the steps do not disappear at temperatures below those required for the formation of the (√31 × √31)*R*±9° reconstruction (cf. Supplementary Fig. 9).

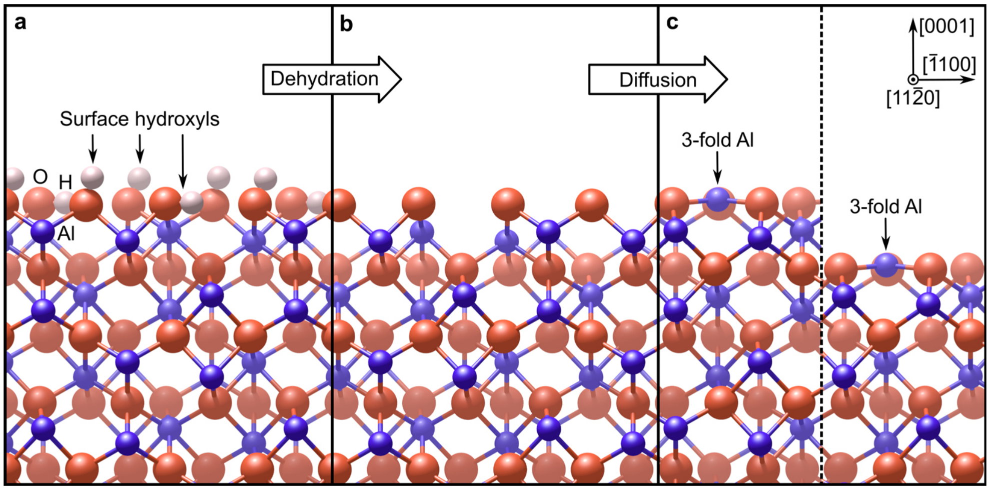


**Supplementary Fig. 13 | Dehydration of an OH-terminated $Al_2O_3$(0001) surface requires mass transport and leads to surface roughening.** (**a**) Fully hydroxylated $Al_2O_3$(0001), similar to the gibbsite structure, is terminated by an OH layer (Supplementary Data 4). Because the $OH^-$ layer carries half the negative charge of an $O^{2-}$ layer in bulk $Al_2O_3$, the surface is non-polar. (**b**) Dehydration removes all H atoms and half of the O atoms from the surface layer. The structure (Supplementary Data 5) is energetically highly unfavorable and shown for illustrative purposes only. Its surface energy after DFT relaxation is 212 meV Å$^{-2}$ (Supplementary Data 6), substantially higher than that of the relaxed (1 × 1) surface, 120 meV Å$^{-2}$ (Supplementary Data 2). (**c**) Conversion of the dehydrated surface into the Al-terminated (1 × 1) structure requires redistribution of both O and Al to the topmost layer and can proceed via diffusion from nearby surface regions. These regions are thereby converted into the same (1 × 1) structure one layer lower, resulting in a rough morphology. Conversely, re-hydration (expected under ambient conditions) also requires mass transport. The facile diffusion of hydroxylated Al in a thin water layer at the surface may lead to different length scales of roughness during hydration and dehydration, increasing roughness over repeated hydration/dehydration cycles.

# Supplementary notes

### Supplementary Note 1 | Additional information for Fig. 2.

The image in panel (a) was acquired with a 900 pm oscillation amplitude and panels (b) and (c) using an oscillation amplitude of 300 pm. Images in panels (a), (b) and (c) were acquired on a sample annealed at ≈ 820 °C in $1 \times 10^{-6}$ mbar $O_2$ for 90 min, and 0 V bias. The image in (g) was recorded with an oscillation amplitude of 900 pm and 1 V bias. The inset of (g) was acquired on a sample annealed at 1300 °C in $1 \times 10^{-7}$ mbar $O_2$ for 60 min using a CuOx-terminated tip at −0.1 V bias with 100 pm oscillation amplitude. Image (d) was acquired on a sample annealed at ≈ 900 °C in $1 \times 10^{-6}$ mbar $O_2$ for 90 min, using a 150 pm amplitude and −0.2 V sample bias.

## Supplementary references


1. Schulze Lammers, B. et al. Benchmarking atomically defined AFM tips for chemical-selective imaging. *Nanoscale* **13**, 13617–13623 (2021).
2. Blöchl, P. E. Projector augmented-wave method. *Phys. Rev. B* **50**, 17953–17979 (1994).
3. Kresse, G. & Joubert, D. From ultrasoft pseudopotentials to the projector augmented-wave method. *Phys. Rev. B* **59**, 1758–1775 (1999).
4. Hütner, J. I. et al. Stoichiometric reconstruction of the $Al_2O_3$(0001) surface. *Science* **385**, 1241–1244 (2024).
5. Balajka, J., Pavelec, J., Komora, M., Schmid, M. & Diebold, U. Apparatus for dosing liquid water in ultrahigh vacuum. *Rev. Sci. Instrum.* **89**, 083906 (2018).
6. Balajka, J. et al. High-affinity adsorption leads to molecularly ordered interfaces on $TiO_2$ in air and solution. *Science* **361**, 786–789 (2018).
7. Henzler, M. Quantitative evaluation of random distributed steps at interfaces and surfaces. *Surf. Sci.* **73**, 240–251 (1978).
