# Supplementary material for: AFM imaging reveals the unreconstructed α‑Al2O3(0001) surface to be inhomogeneous and rough
Source: Nat Commun. 2026 May 27;17:4692. doi: 10.1038/s41467-026-73690-0 (PMC13216582; doi:10.1038/s41467-026-73690-0)
Supplement: Supplementary file 2 — Description of Additional Supplementary Files [file 41467_2026_73690_MOESM2_ESM.pdf]

Description of Additional Supplementary Files

Supplementary Data 1 | Structure model of the bulk-truncated Alterminated $Al2O3(0001)$-(1 × 1) surface shown in Fig. 1b.

Supplementary Data 2 | DFT-relaxed structure model of the Al-terminated $Al2O3(0001)$-(1 × 1) surface (Fig. 1c,e).

Supplementary Data 3 | DFT-relaxed structure model of the $Al2O3(0001)$ surface with steps in a (2 × 6) unit cell (Supplementary Fig. 12).

Supplementary Data 4 | DFT-relaxed structure model of the hydroxylated $Al2O3(0001)$ surface (Supplementary Fig. 13a).

Supplementary Data 5 | Structure model of the dehydrated $Al2O3(0001)$ surface (Fig. 13b).

Supplementary Data 6 | DFT-relaxed structure model of the dehydrated $Al2O3(0001)$ surface (Supplementary Fig. 13b)

Supplementary Data 7 | Raw experimental data corresponding to the nc-AFM, LEED, and XPS measurements presented in the manuscript and Supplementary Information.
