# Supplementary figures and images for: AFM imaging reveals the unreconstructed α‑Al2O3(0001) surface to be inhomogeneous and rough
Source: Nat Commun. 2026 May 27;17:4692. doi: 10.1038/s41467-026-73690-0 (PMC13216582; doi:10.1038/s41467-026-73690-0)

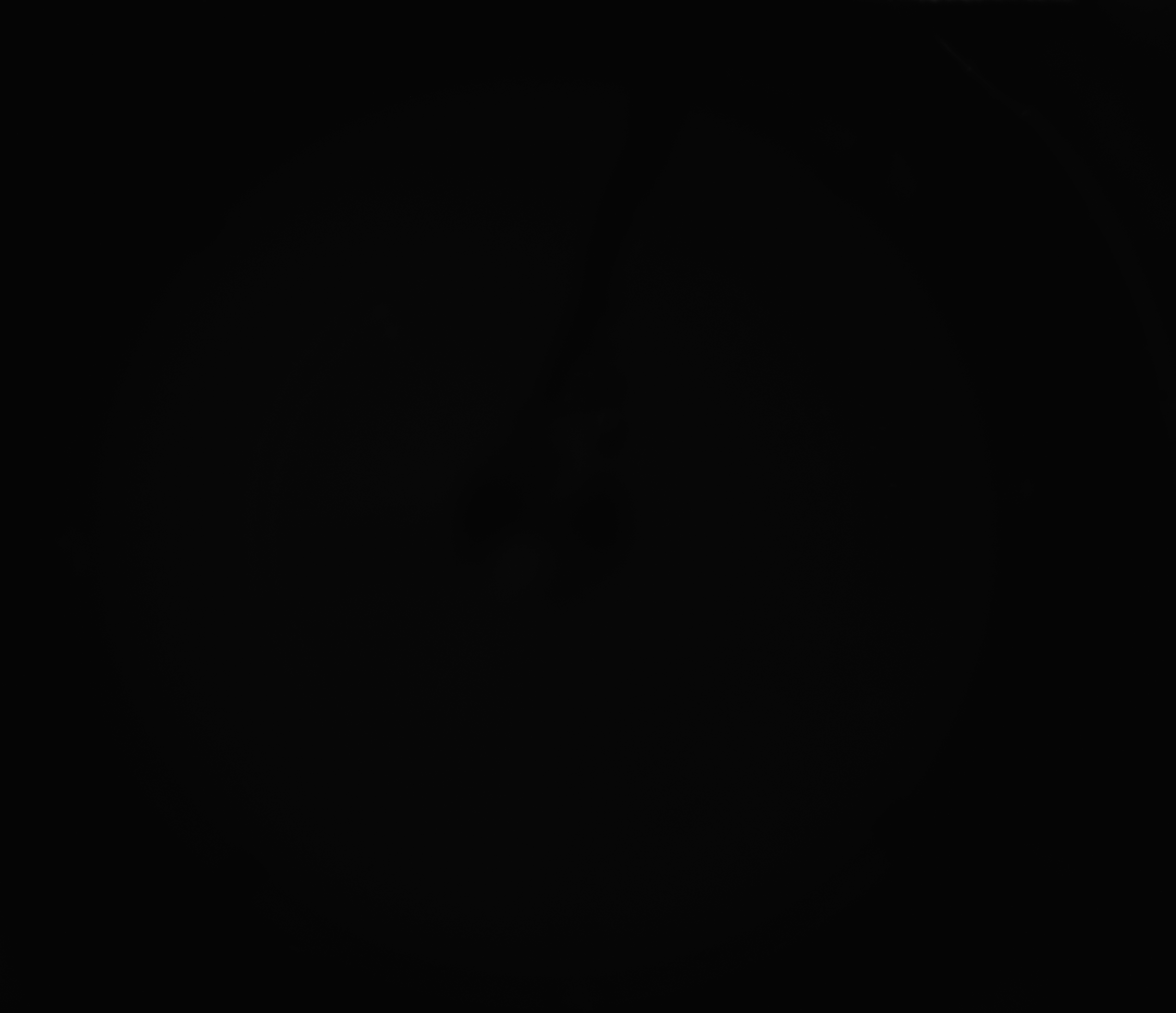

Supplement: Supplementary file 9 — Supplementary Data 7 [file 41467_2026_73690_MOESM9_ESM.zip › raw data/FigS02LEED_transition-to-rec/1020C_8.3A_20230728_E1/SFig.2_1020C_8.3A_20230728_E1_000eV_2-3A_0kV_dark.tiff]

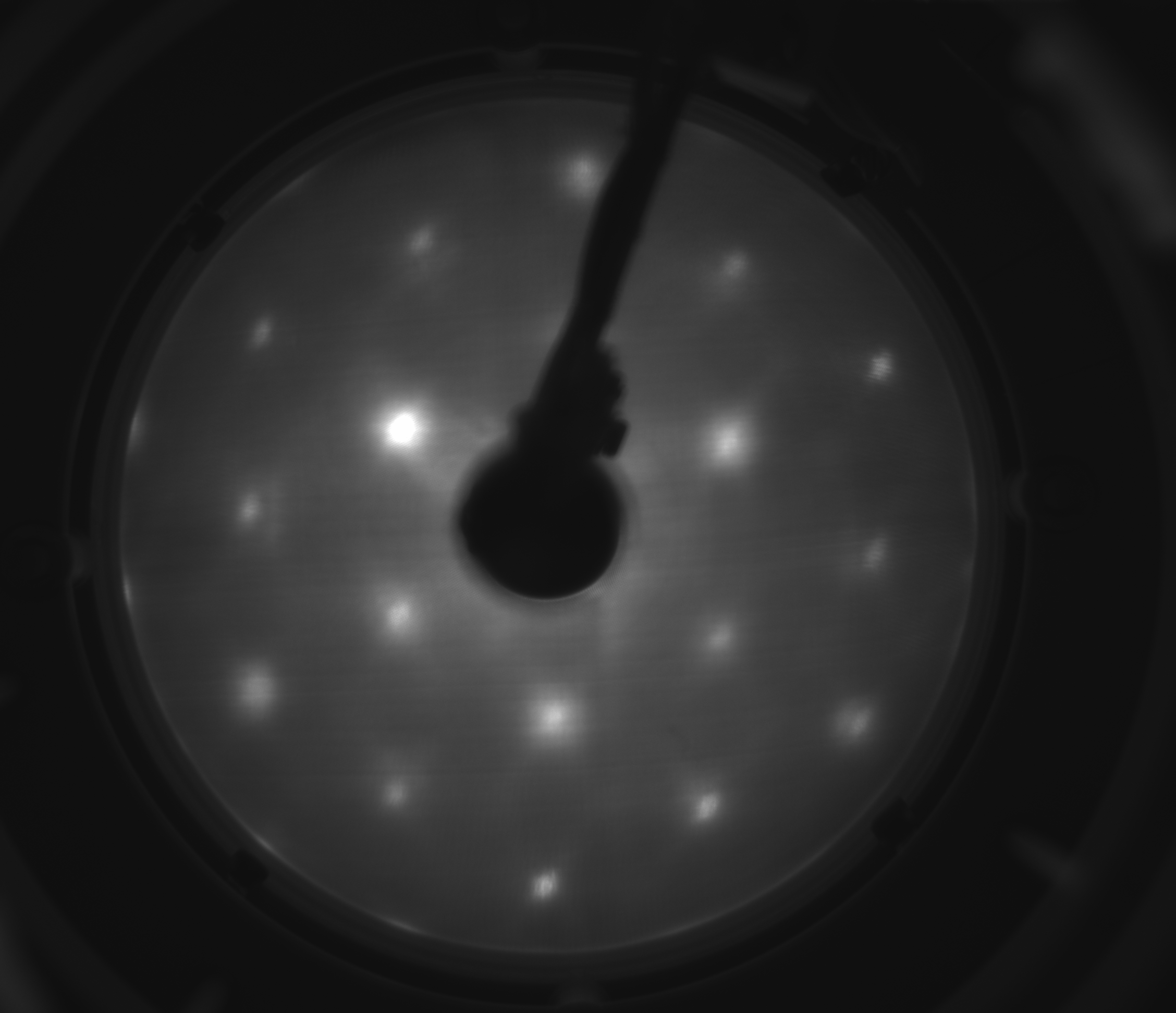

Supplement: Supplementary file 9 — Supplementary Data 7 [file 41467_2026_73690_MOESM9_ESM.zip › raw data/FigS02LEED_transition-to-rec/1020C_8.3A_20230728_E1/SFig.2_1020C_8.3A_20230728_E1_120eV_2-3A_6kV.tiff]

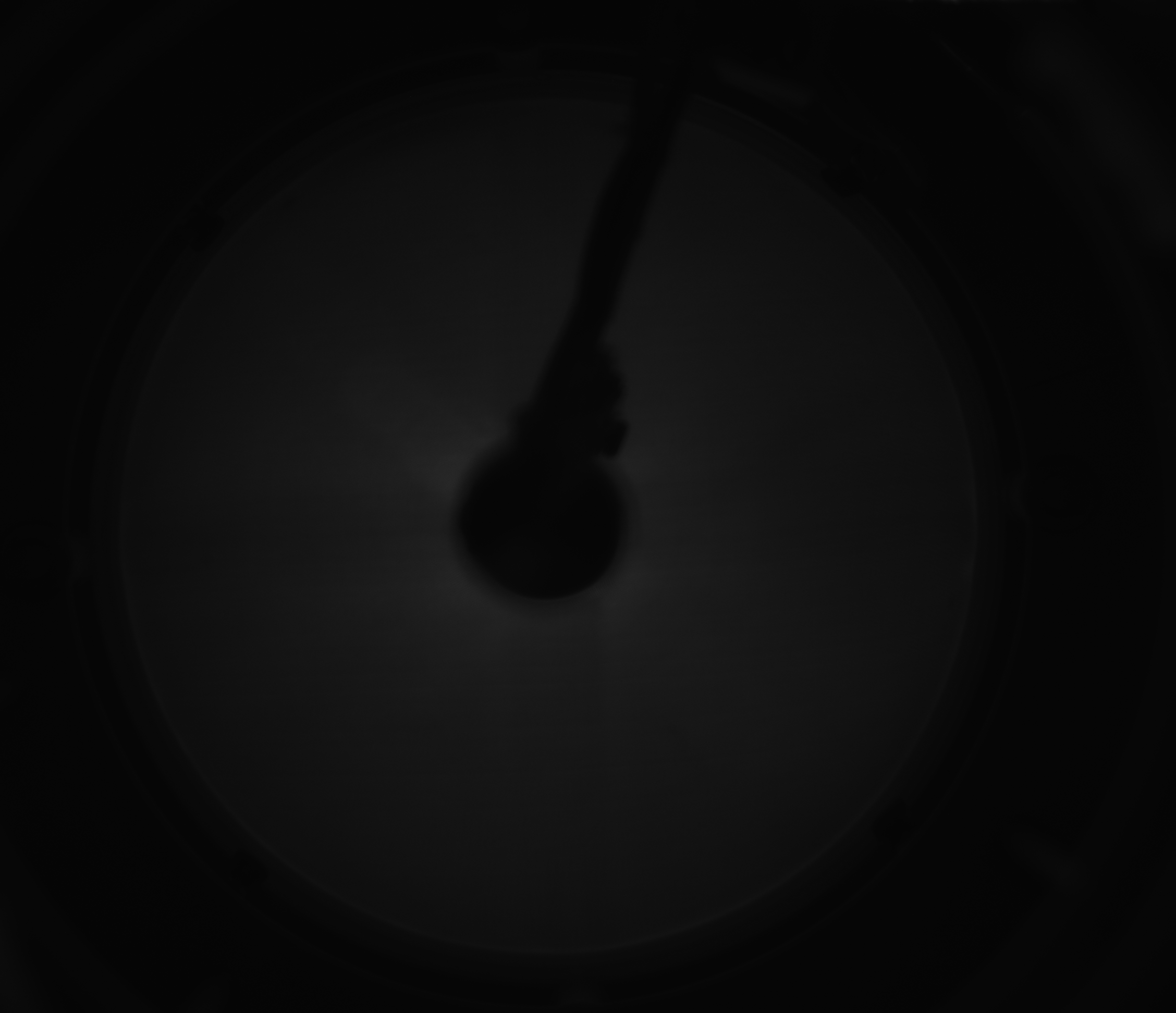

Supplement: Supplementary file 9 — Supplementary Data 7 [file 41467_2026_73690_MOESM9_ESM.zip › raw data/FigS02LEED_transition-to-rec/1020C_8.3A_20230728_E1/SFig.2_1020C_8.3A_20230728_E1_120eV_2-3A_6kV_flat.tiff]

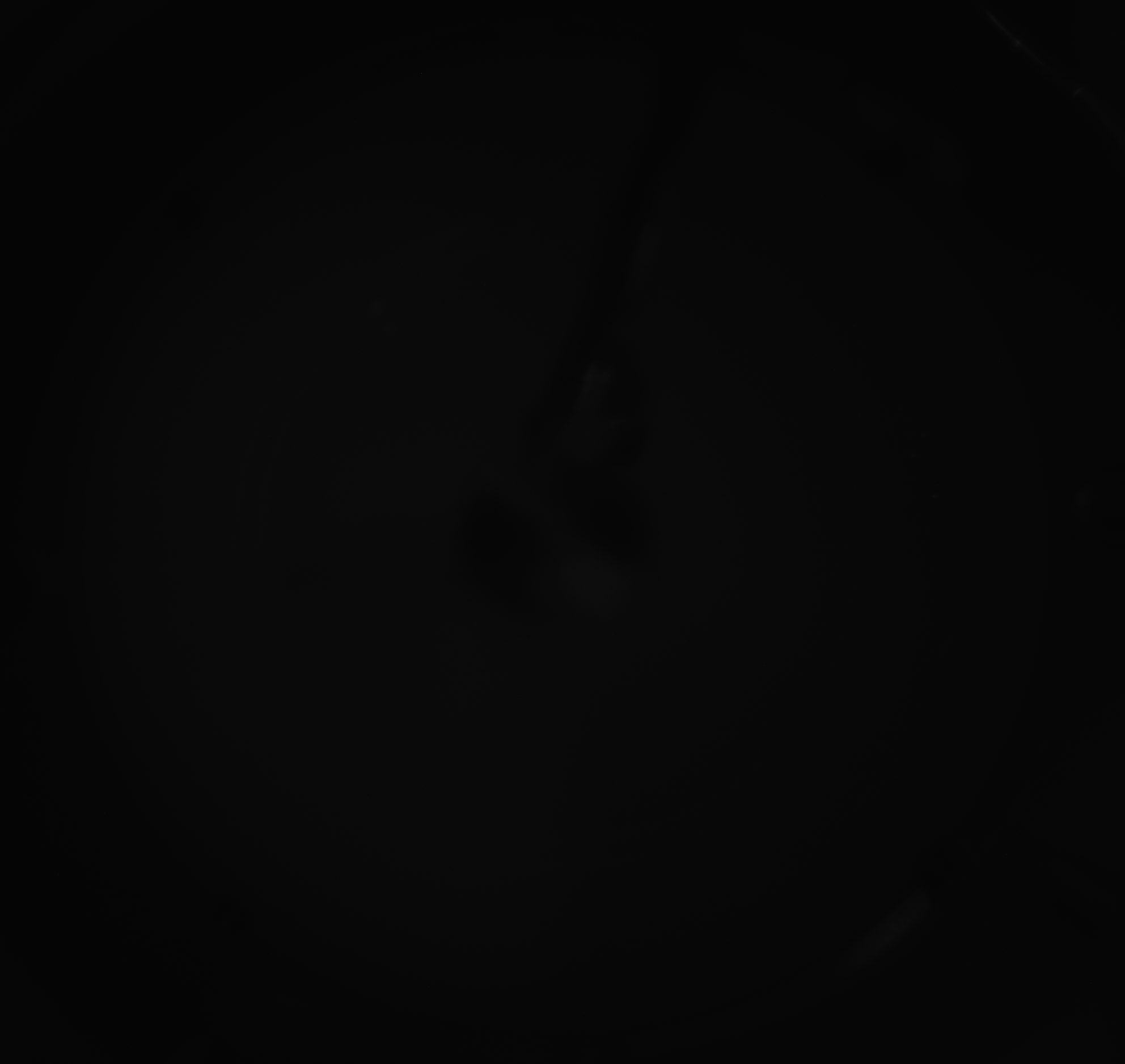

Supplement: Supplementary file 9 — Supplementary Data 7 [file 41467_2026_73690_MOESM9_ESM.zip › raw data/FigS02LEED_transition-to-rec/1050C_8.5A_20230728_E1/SFig.2_1050C_8.5A_20230728_E1_000eV_2-3A_6kV_dark.tiff]

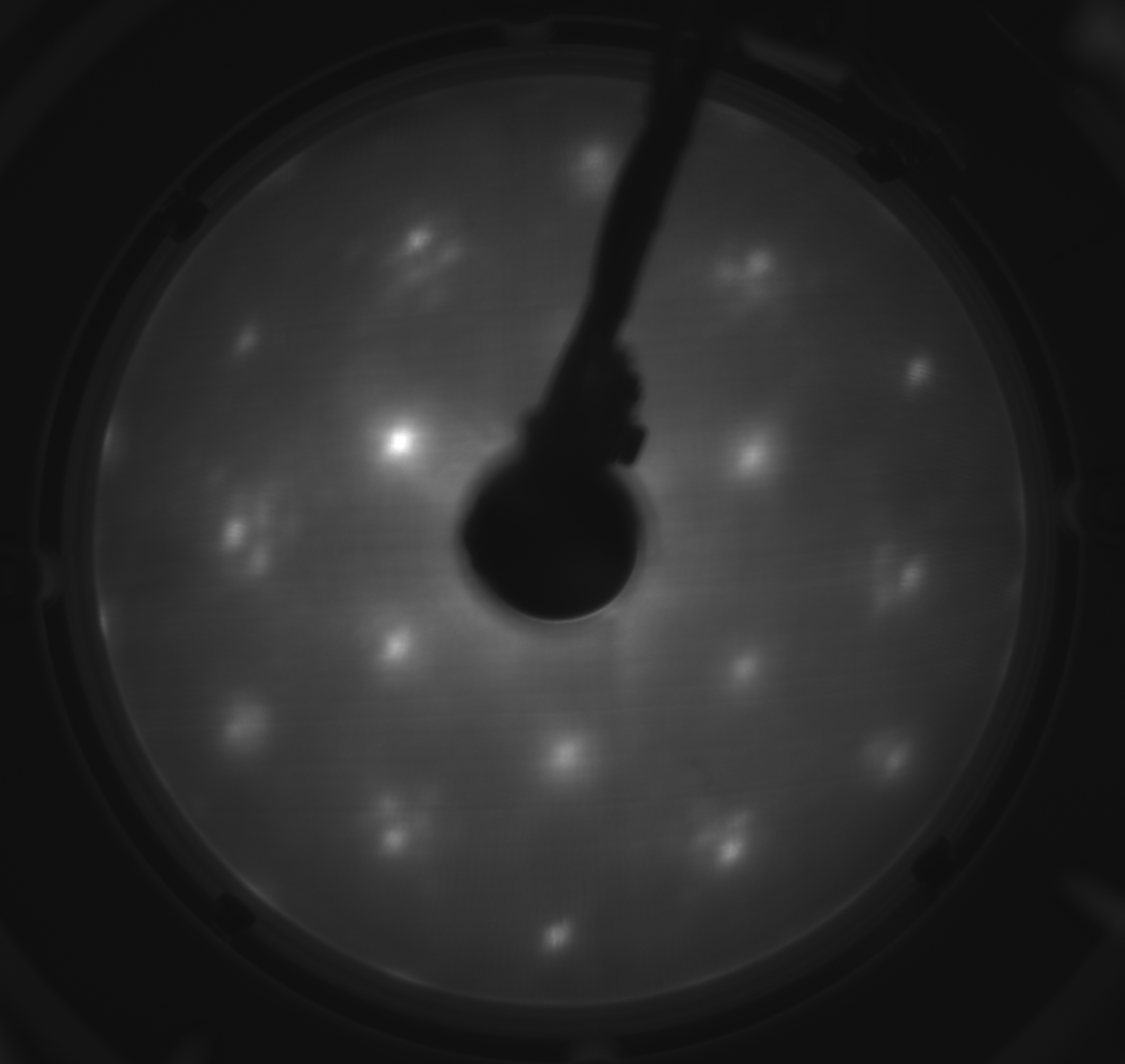

Supplement: Supplementary file 9 — Supplementary Data 7 [file 41467_2026_73690_MOESM9_ESM.zip › raw data/FigS02LEED_transition-to-rec/1050C_8.5A_20230728_E1/SFig.2_1050C_8.5A_20230728_E1_120eV_2-3A_6kV.tiff]

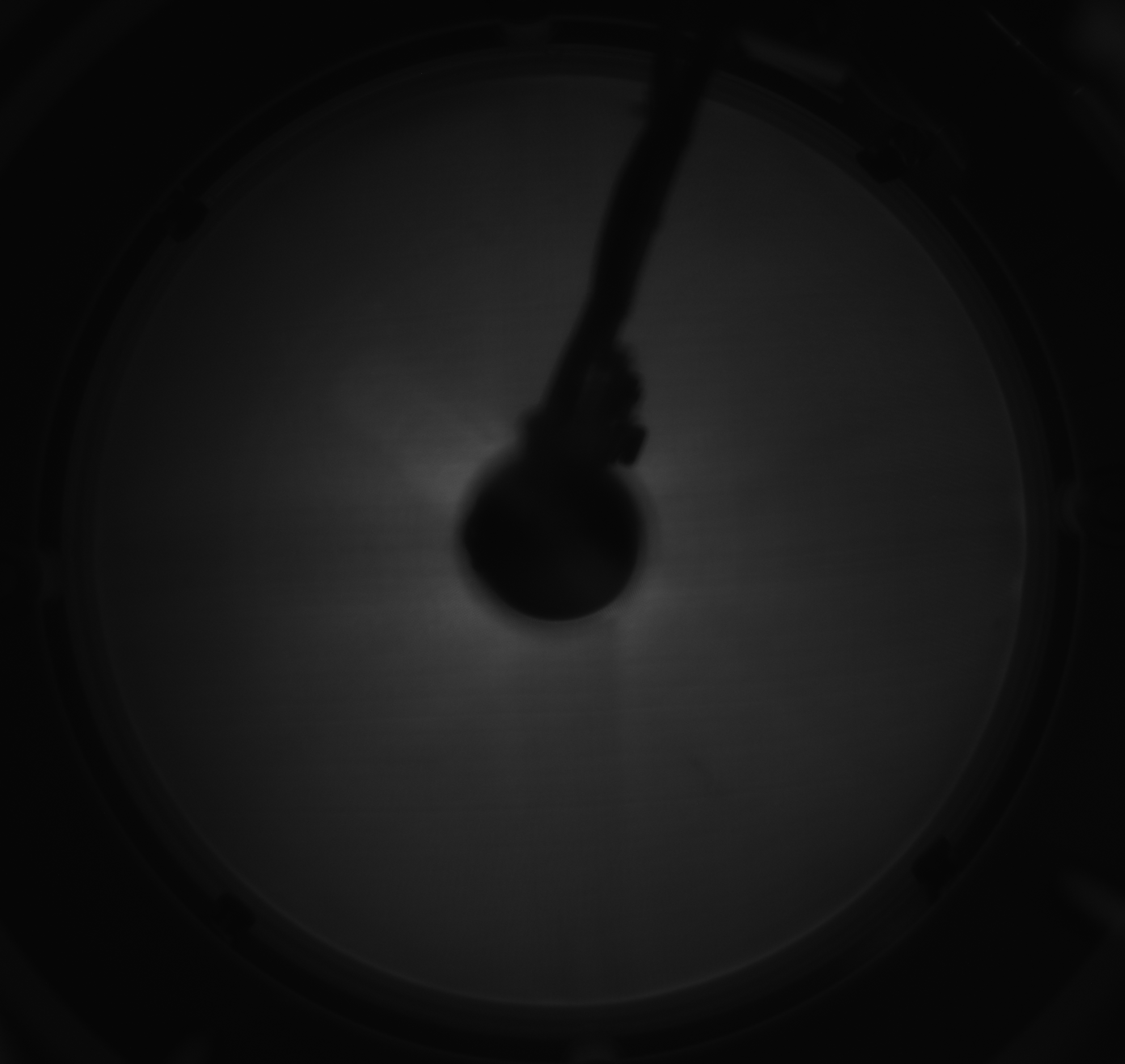

Supplement: Supplementary file 9 — Supplementary Data 7 [file 41467_2026_73690_MOESM9_ESM.zip › raw data/FigS02LEED_transition-to-rec/1050C_8.5A_20230728_E1/SFig.2_1050C_8.5A_20230728_E1_120eV_2-3A_6kV_flat.tiff]

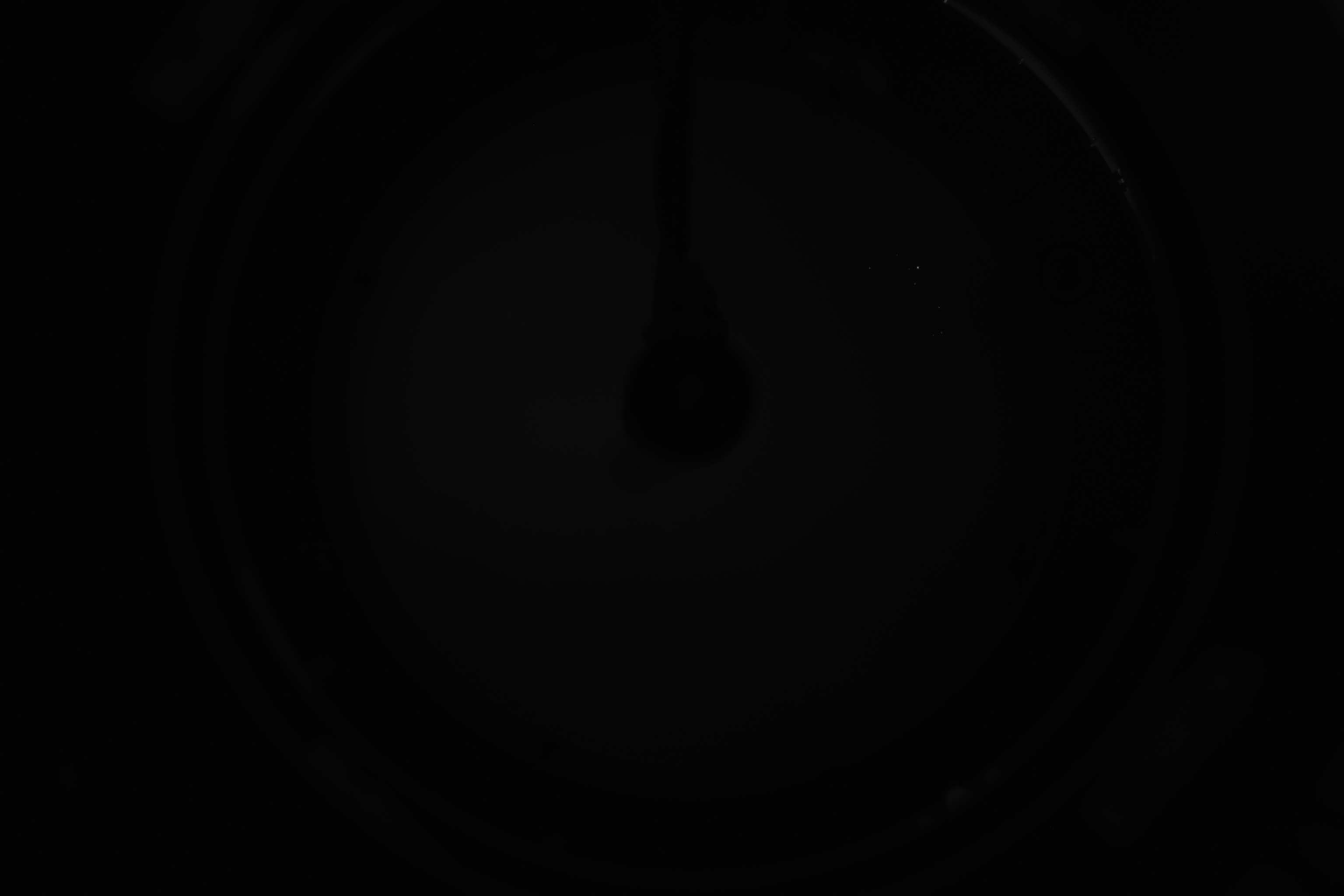

Supplement: Supplementary file 9 — Supplementary Data 7 [file 41467_2026_73690_MOESM9_ESM.zip › raw data/FigS02LEED_transition-to-rec/1300C_10A_20240422_B3/SFig.2_1300C_10A_20240422_B3_120eV_2-3A_0kV_dark.tiff]

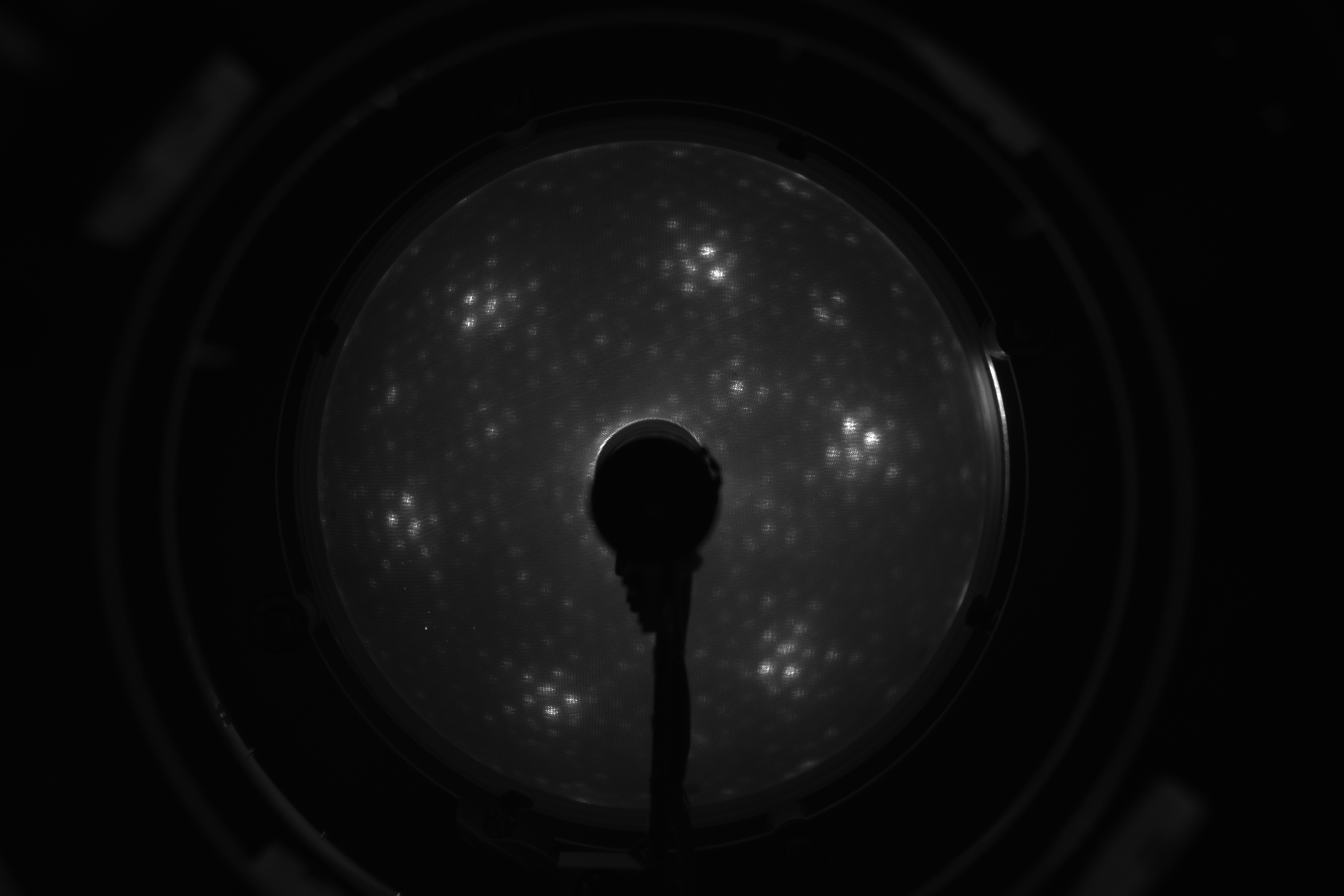

Supplement: Supplementary file 9 — Supplementary Data 7 [file 41467_2026_73690_MOESM9_ESM.zip › raw data/FigS02LEED_transition-to-rec/1300C_10A_20240422_B3/SFig.2_1300C_10A_20240422_B3_120eV_2-3A_6kV.tiff]

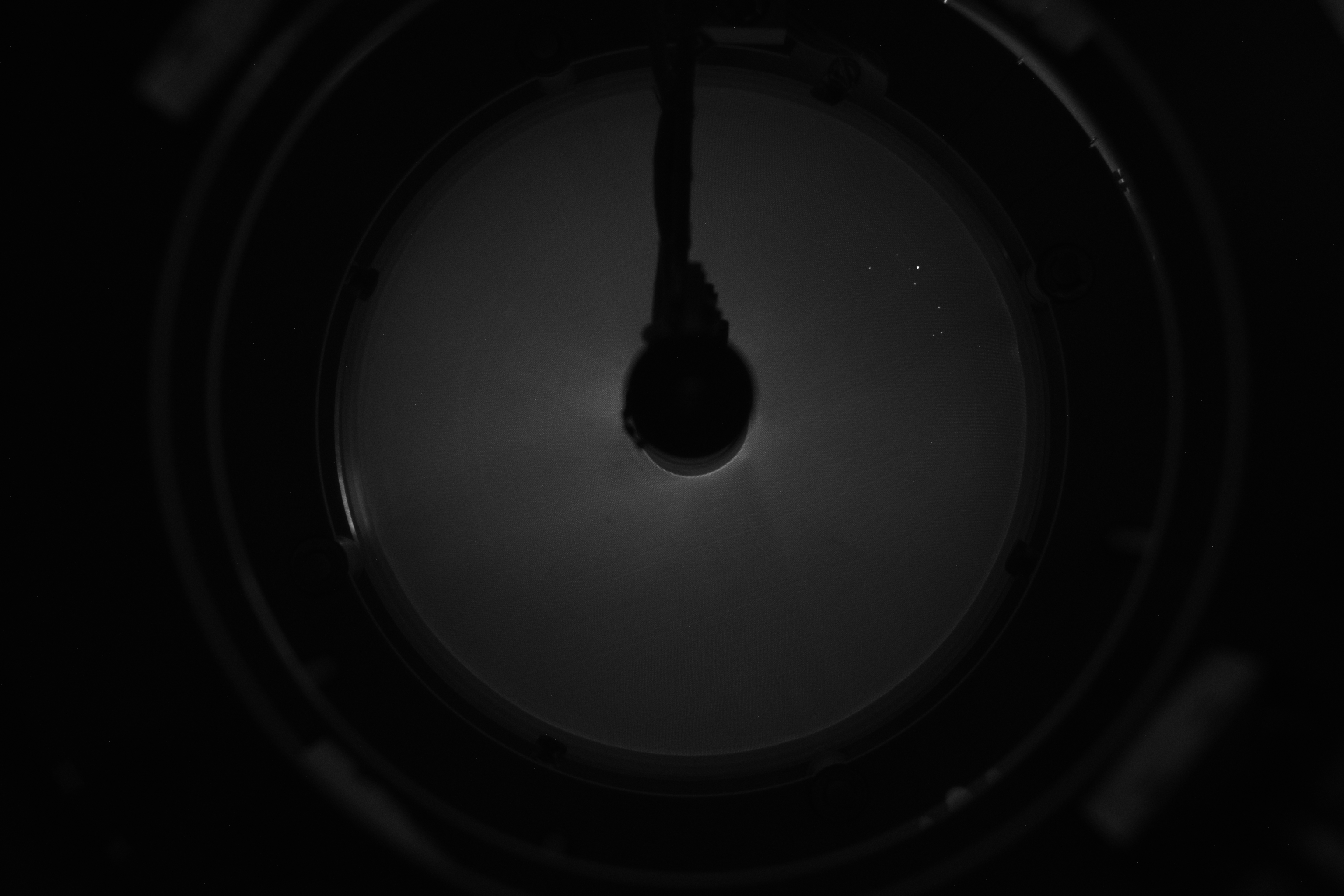

Supplement: Supplementary file 9 — Supplementary Data 7 [file 41467_2026_73690_MOESM9_ESM.zip › raw data/FigS02LEED_transition-to-rec/1300C_10A_20240422_B3/SFig.2_1300C_10A_20240422_B3_150eV_2-3A_6kVflat.tiff]

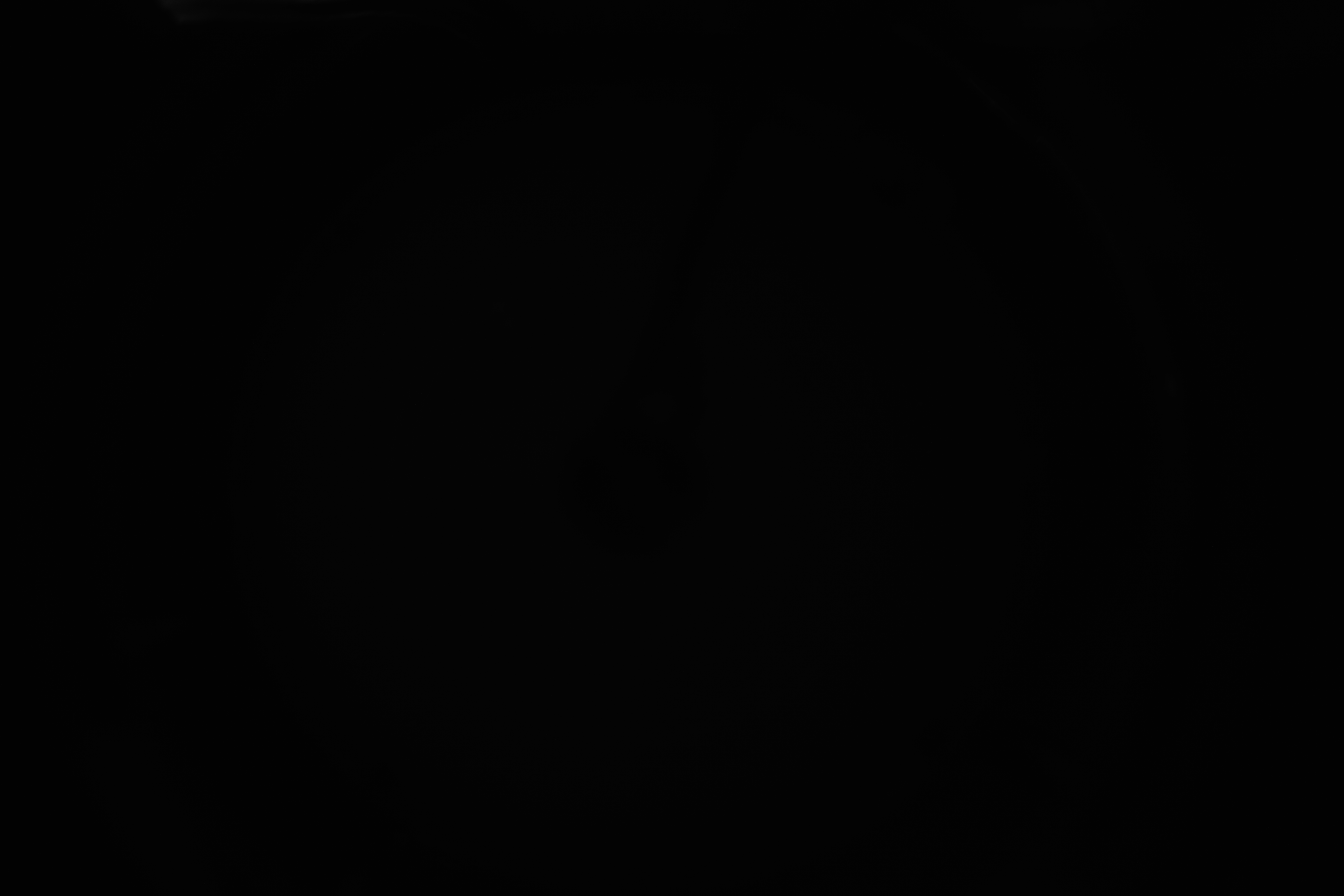

Supplement: Supplementary file 9 — Supplementary Data 7 [file 41467_2026_73690_MOESM9_ESM.zip › raw data/FigS02LEED_transition-to-rec/900C_7.5A_20230727_E1/SFig.2_900C_7.5A_20230727_E1_000eV_2-3A_6kV_dark.tiff]

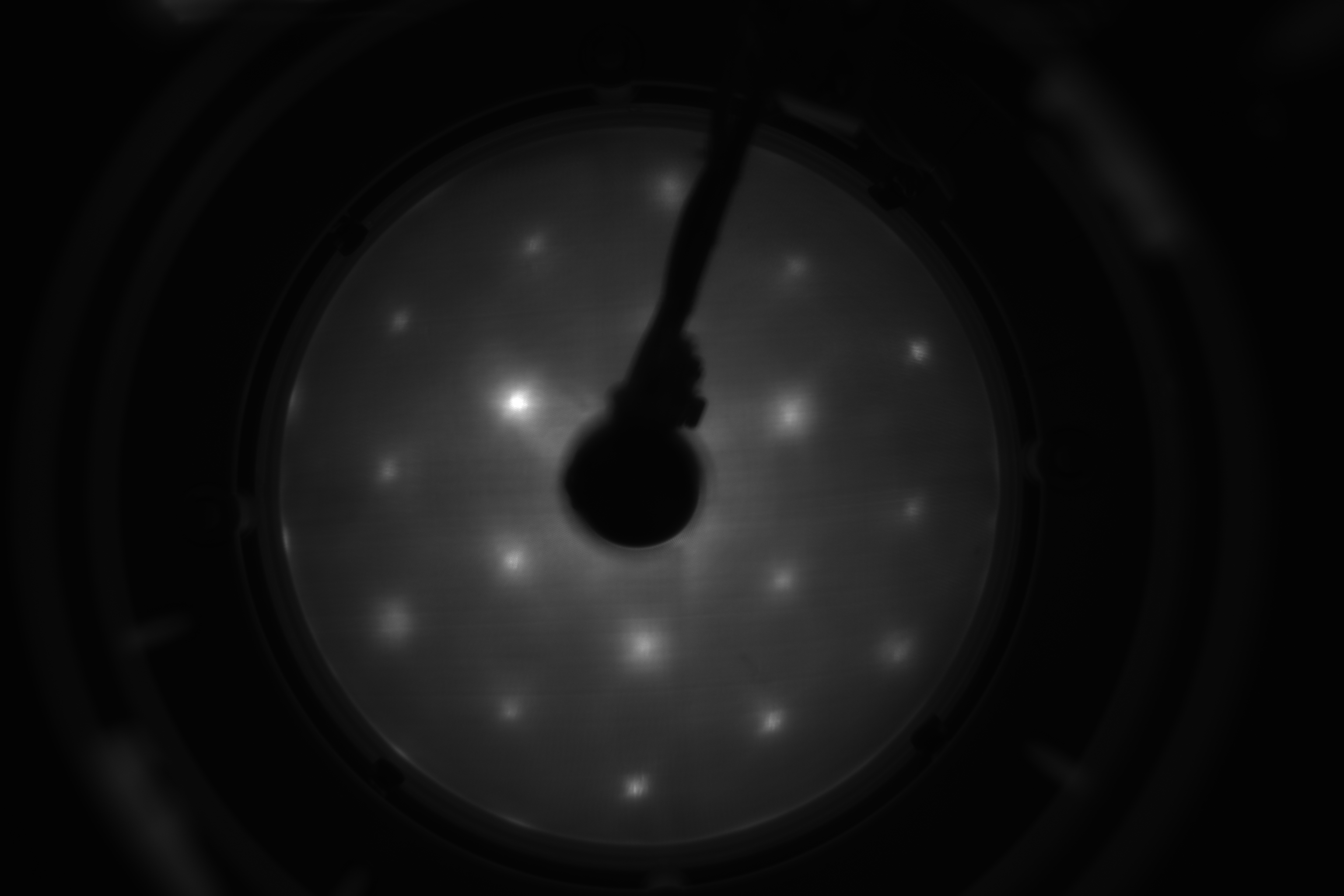

Supplement: Supplementary file 9 — Supplementary Data 7 [file 41467_2026_73690_MOESM9_ESM.zip › raw data/FigS02LEED_transition-to-rec/900C_7.5A_20230727_E1/SFig.2_900C_7.5A_20230727_E1_120eV_2-3A_6kV.tiff]

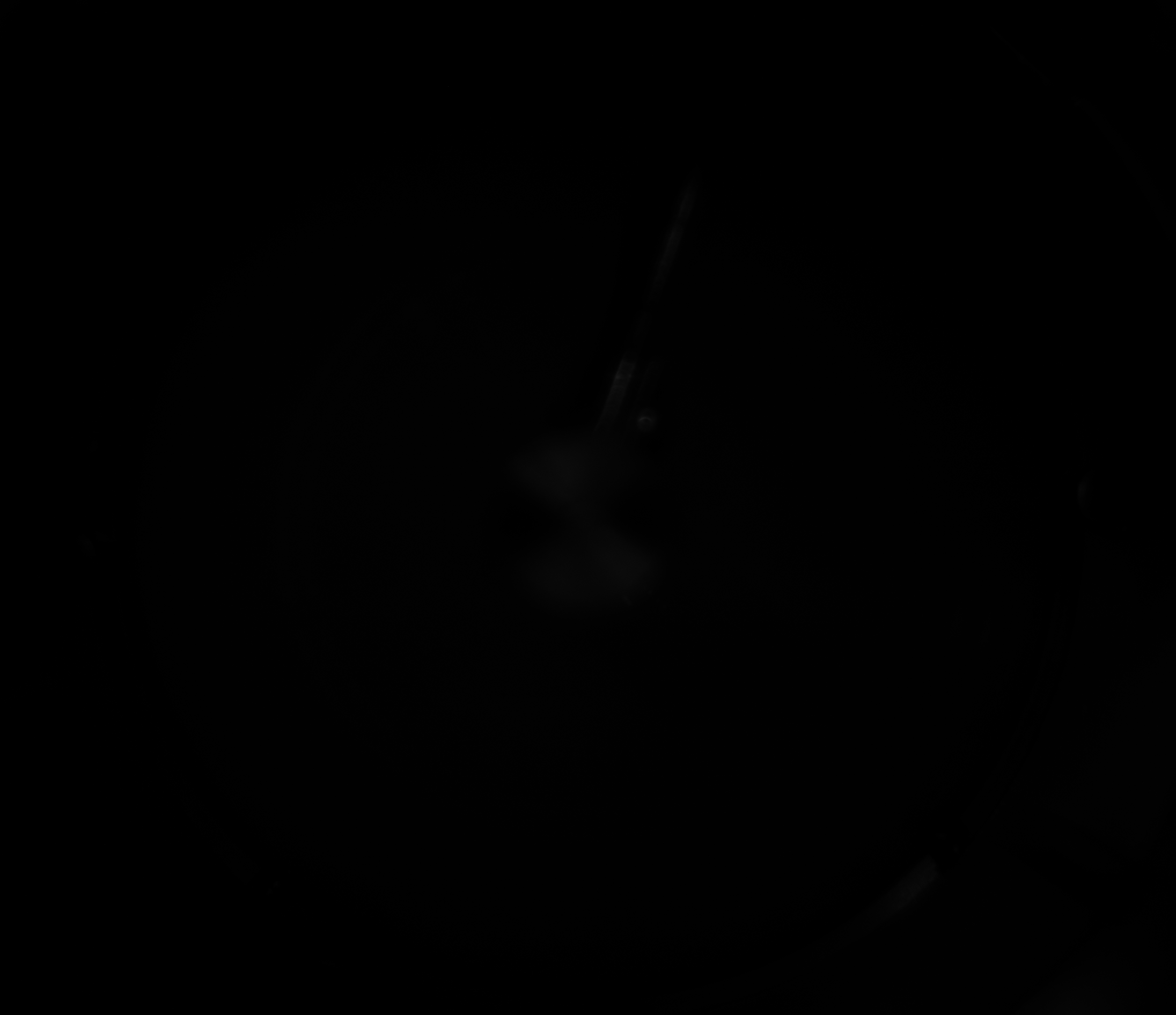

Supplement: Supplementary file 9 — Supplementary Data 7 [file 41467_2026_73690_MOESM9_ESM.zip › raw data/FigS02LEED_transition-to-rec/980C_8.1A_20230727_E1/SFig.2_980C_8.1A_20230727_E1_000eV_2-3A_6kV_dark.tiff]

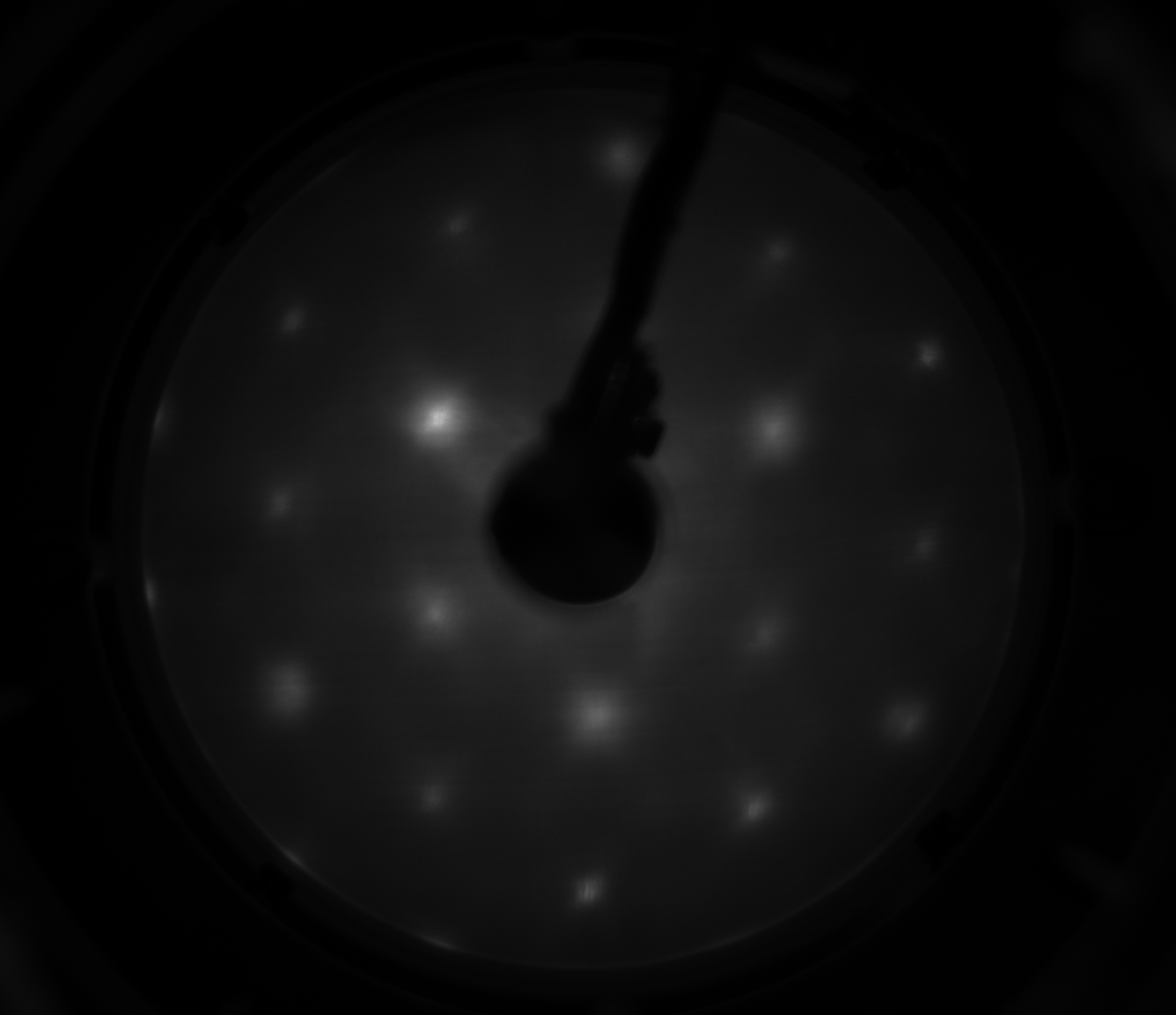

Supplement: Supplementary file 9 — Supplementary Data 7 [file 41467_2026_73690_MOESM9_ESM.zip › raw data/FigS02LEED_transition-to-rec/980C_8.1A_20230727_E1/SFig.2_980C_8.1A_20230727_E1_120eV_2-3A_6kV.tiff]

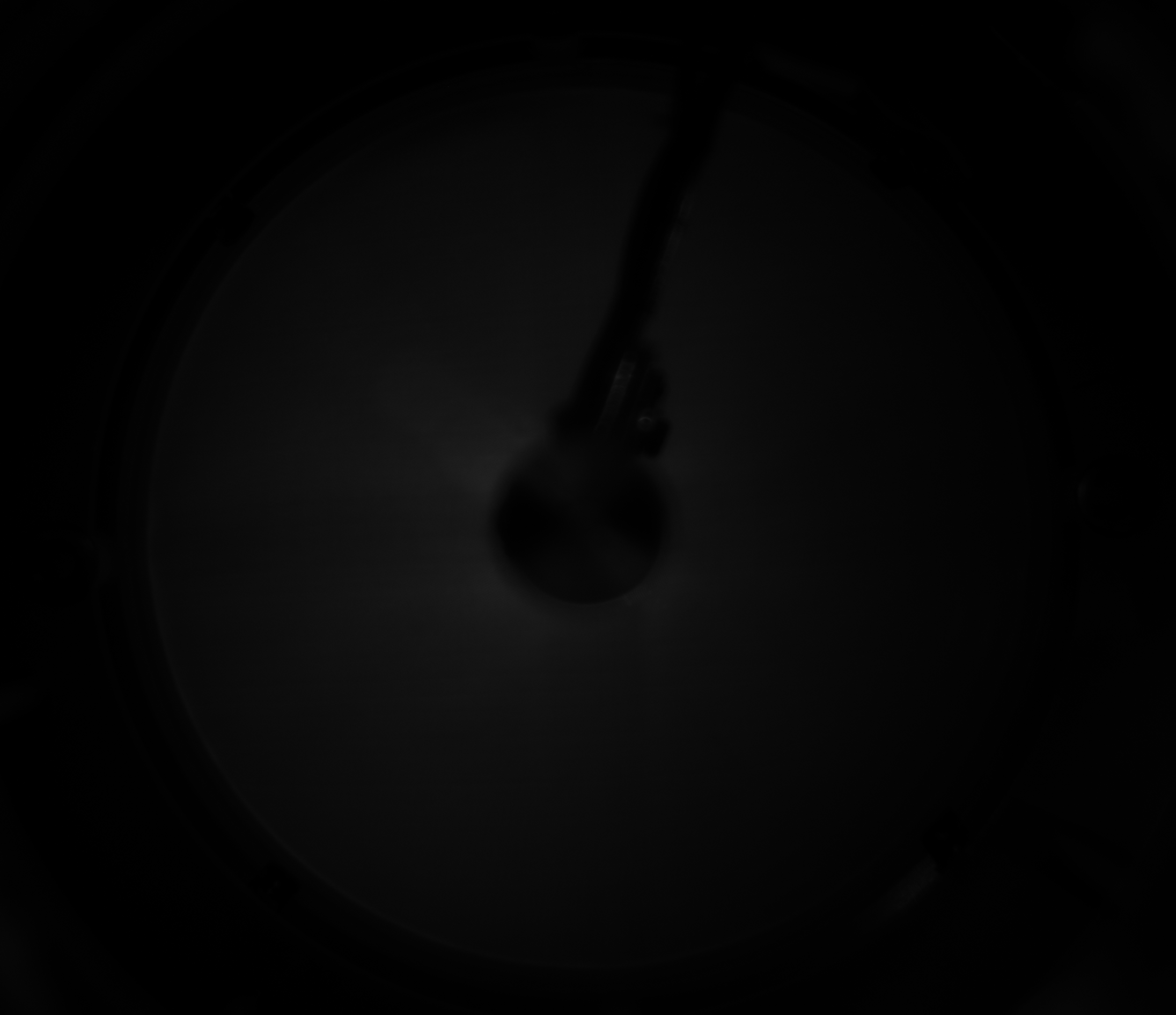

Supplement: Supplementary file 9 — Supplementary Data 7 [file 41467_2026_73690_MOESM9_ESM.zip › raw data/FigS02LEED_transition-to-rec/980C_8.1A_20230727_E1/SFig.2_980C_8.1A_20230727_E1_120eV_2-3A_6kV_flat.tiff]

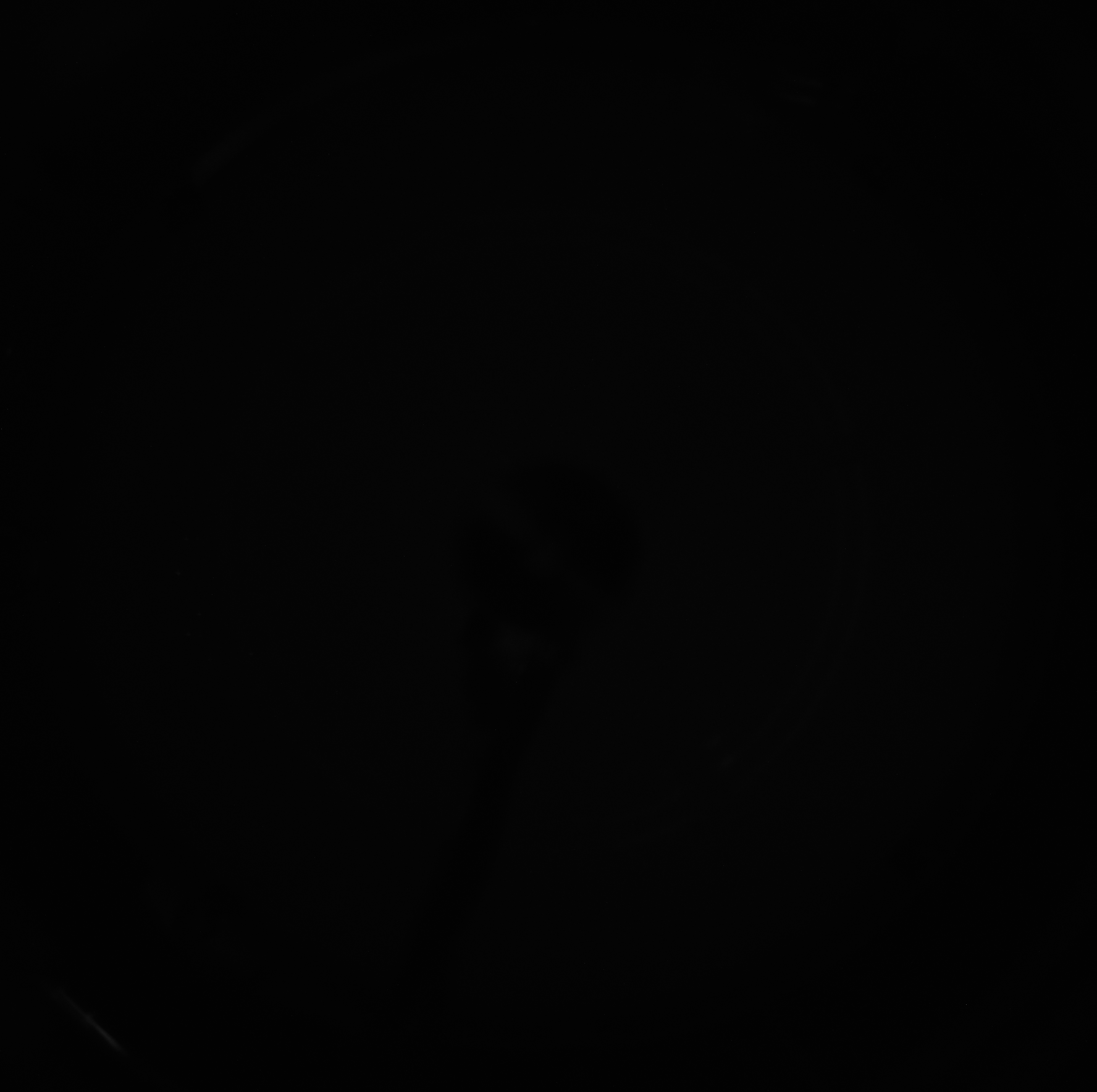

Supplement: Supplementary file 9 — Supplementary Data 7 [file 41467_2026_73690_MOESM9_ESM.zip › raw data/FigS10_LEED_rec/Fig.S10a-left_LEED_A5_annealed-UHV/SFig.10a_20231124_A5_000eV_Screen6kV_2.3A_dark.tiff]

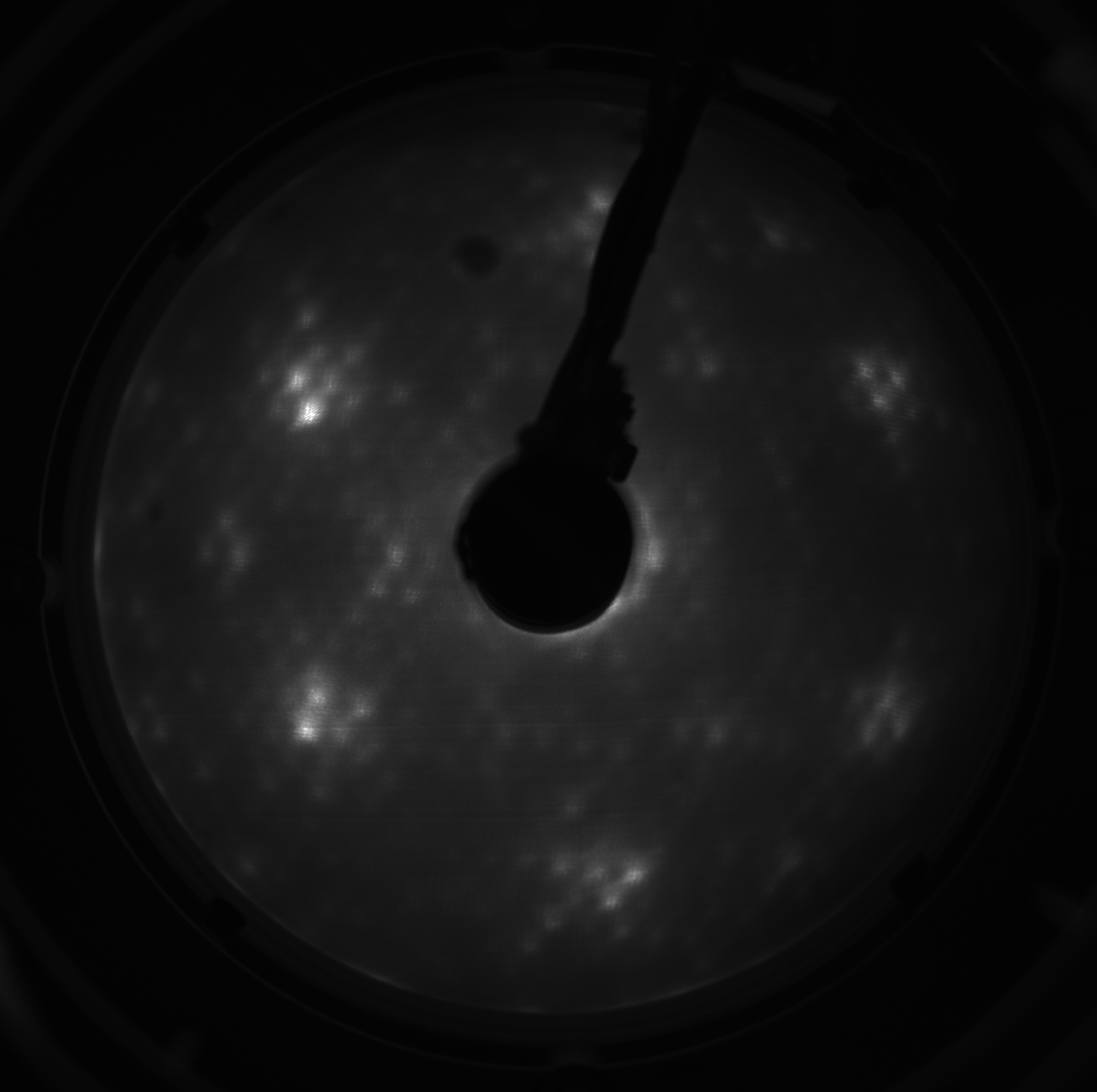

Supplement: Supplementary file 9 — Supplementary Data 7 [file 41467_2026_73690_MOESM9_ESM.zip › raw data/FigS10_LEED_rec/Fig.S10a-left_LEED_A5_annealed-UHV/SFig.10a_20231124_A5_120eV_Screen6kV_2.3A.tiff]

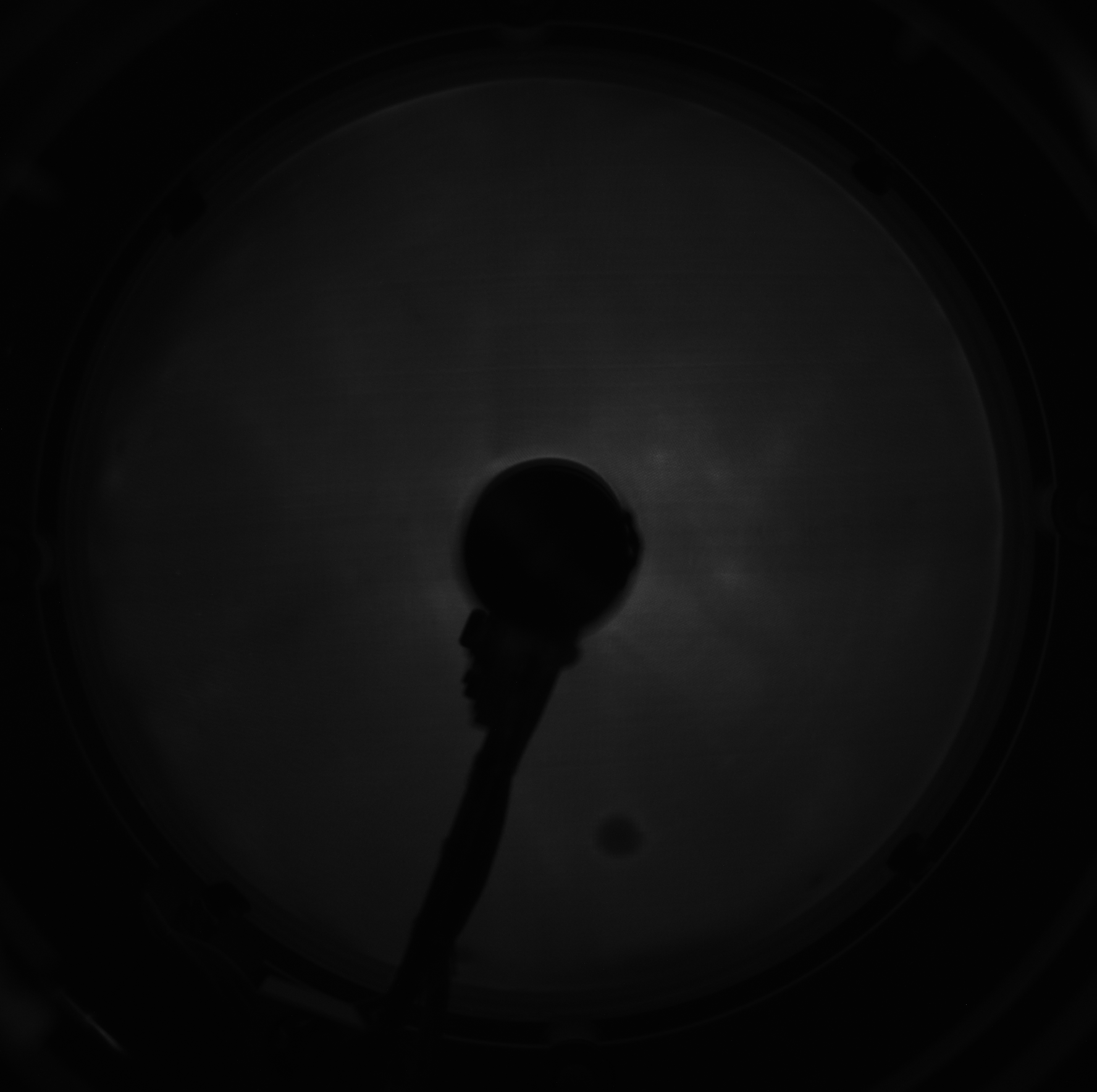

Supplement: Supplementary file 9 — Supplementary Data 7 [file 41467_2026_73690_MOESM9_ESM.zip › raw data/FigS10_LEED_rec/Fig.S10a-left_LEED_A5_annealed-UHV/SFig.10a_20231124_A5_120eV_Screen6kV_2.3A_flat.tiff]

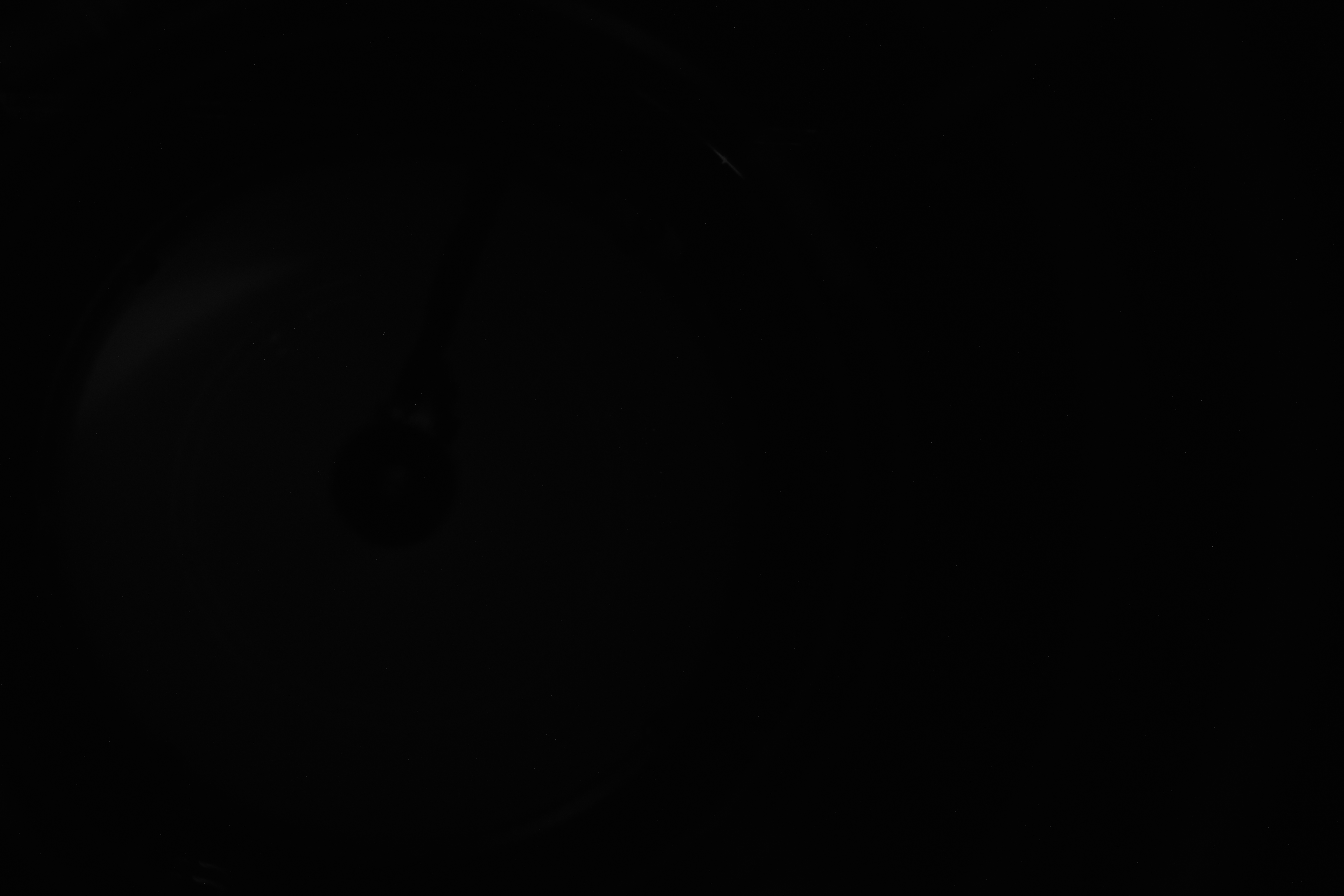

Supplement: Supplementary file 9 — Supplementary Data 7 [file 41467_2026_73690_MOESM9_ESM.zip › raw data/FigS10_LEED_rec/Fig.S10a-right_LEED_A5_annealed-in-O2/SFig.10a-right_20231124_A5_O2_000eV_Screen6kV_2.3A_dark.tiff]

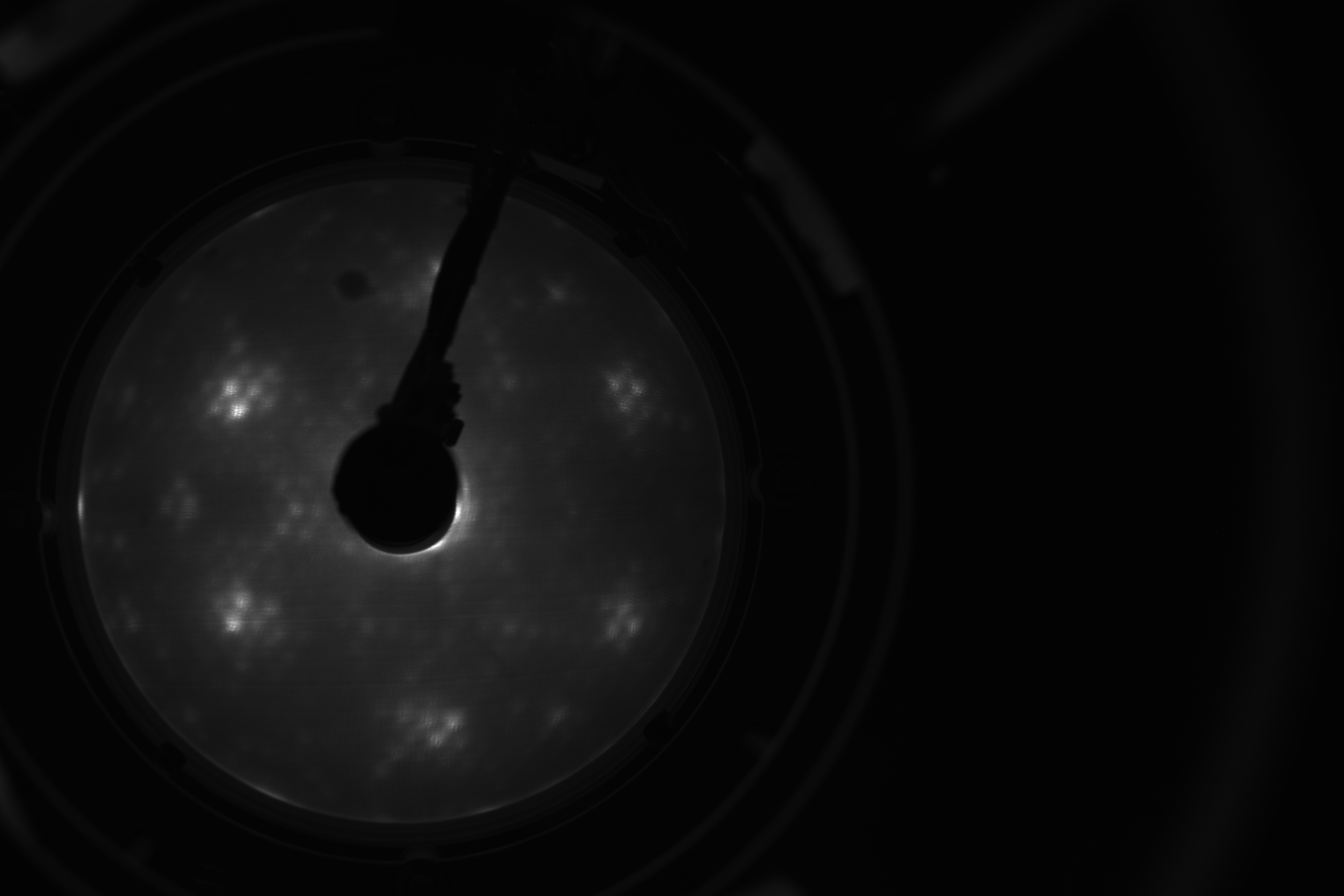

Supplement: Supplementary file 9 — Supplementary Data 7 [file 41467_2026_73690_MOESM9_ESM.zip › raw data/FigS10_LEED_rec/Fig.S10a-right_LEED_A5_annealed-in-O2/SFig.10a-right_20231124_A5_O2_120eV_Screen6kV_2.3A.tiff]

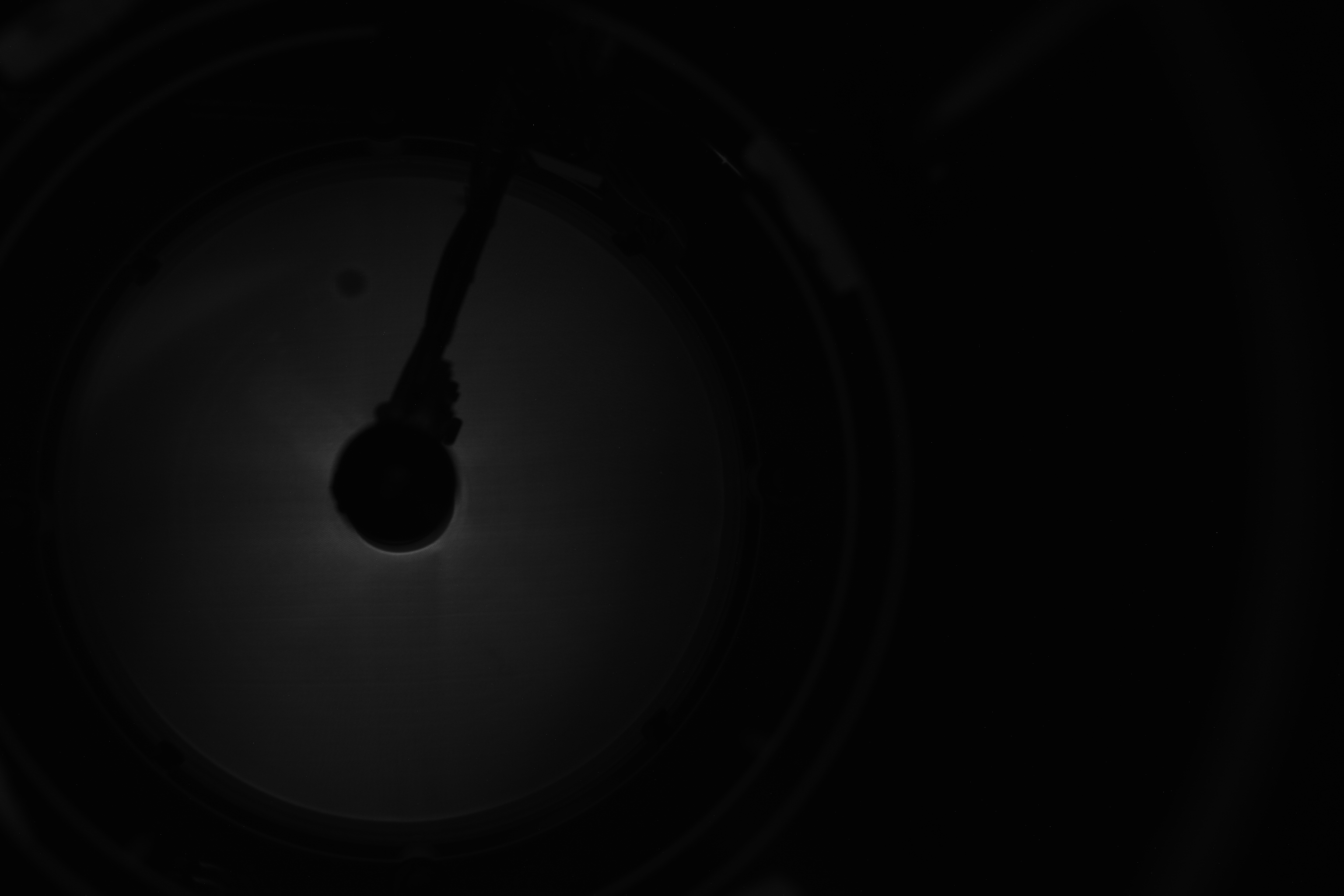

Supplement: Supplementary file 9 — Supplementary Data 7 [file 41467_2026_73690_MOESM9_ESM.zip › raw data/FigS10_LEED_rec/Fig.S10a-right_LEED_A5_annealed-in-O2/SFig.10a-right_20231124_A5_O2_120eV_Screen6kV_2.3A_flat.tiff]

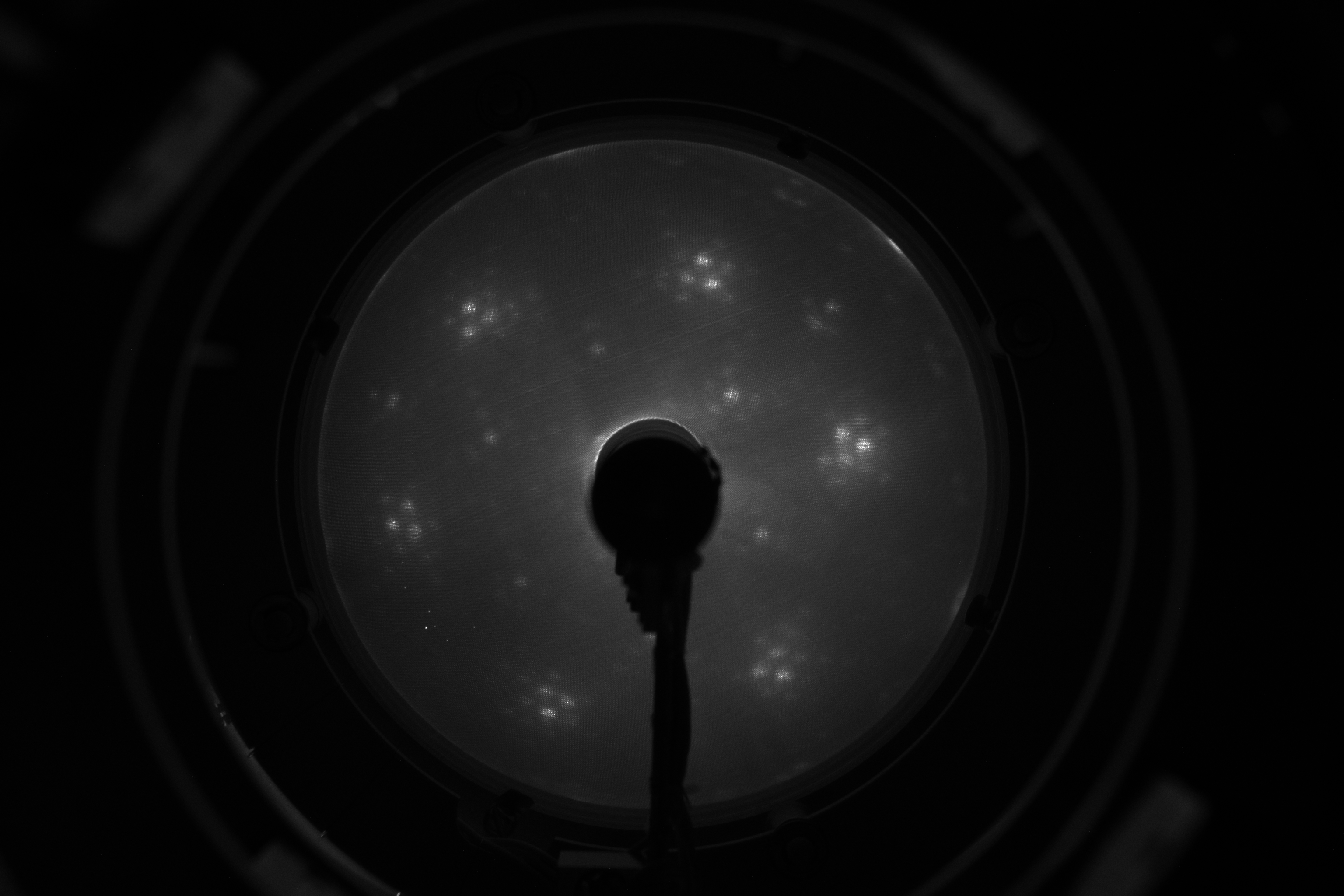

Supplement: Supplementary file 9 — Supplementary Data 7 [file 41467_2026_73690_MOESM9_ESM.zip › raw data/FigS10_LEED_rec/Fig.S10b-right_LEED_B3_after-drop/SFig.10b-right_20240425_B3_H2O_120eV_2-3A_6kV.tiff]

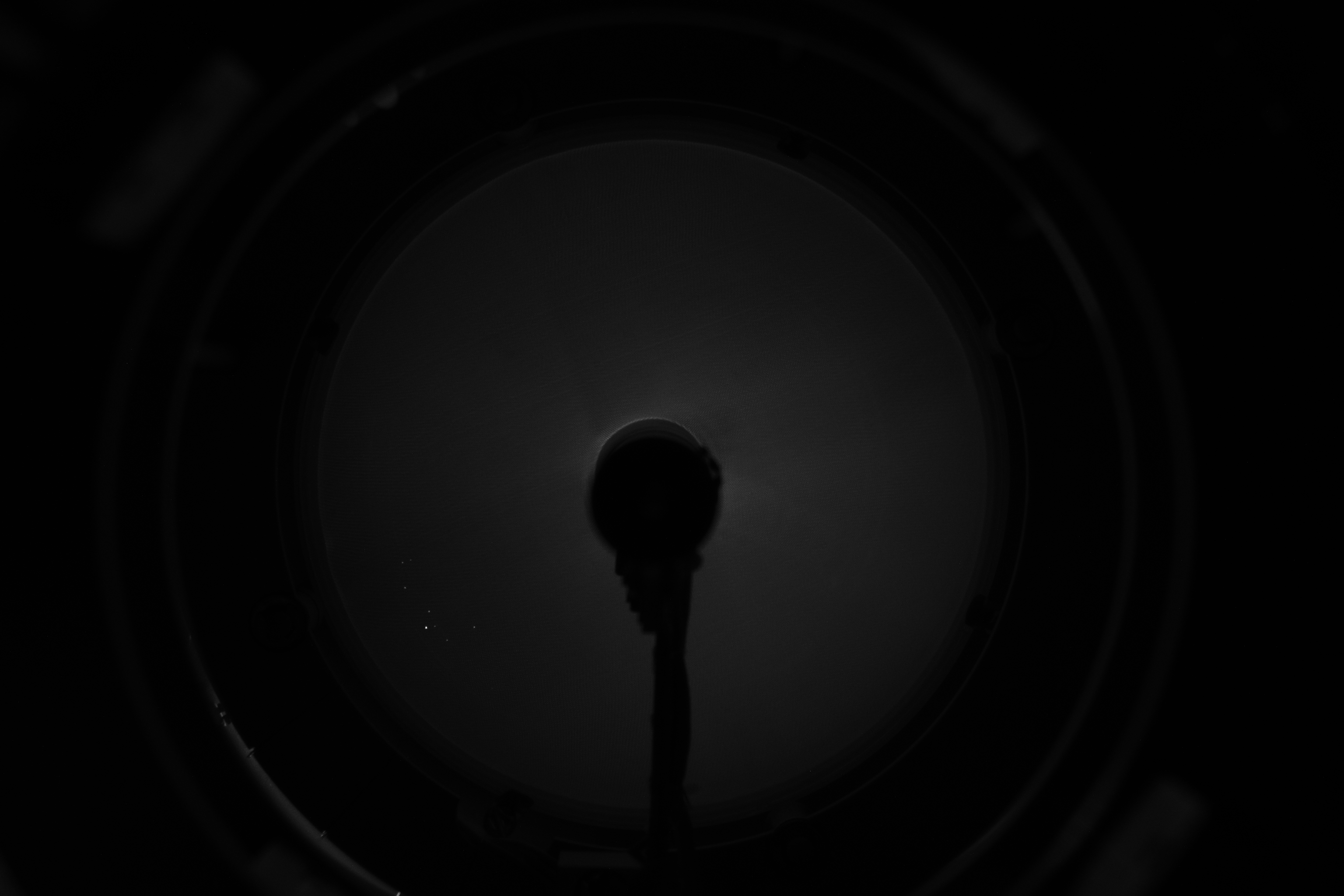

Supplement: Supplementary file 9 — Supplementary Data 7 [file 41467_2026_73690_MOESM9_ESM.zip › raw data/FigS10_LEED_rec/Fig.S10b-right_LEED_B3_after-drop/SFig.10b-right_20240425_B3_H2O_120eV_2-3A_6kV_flat.tiff]

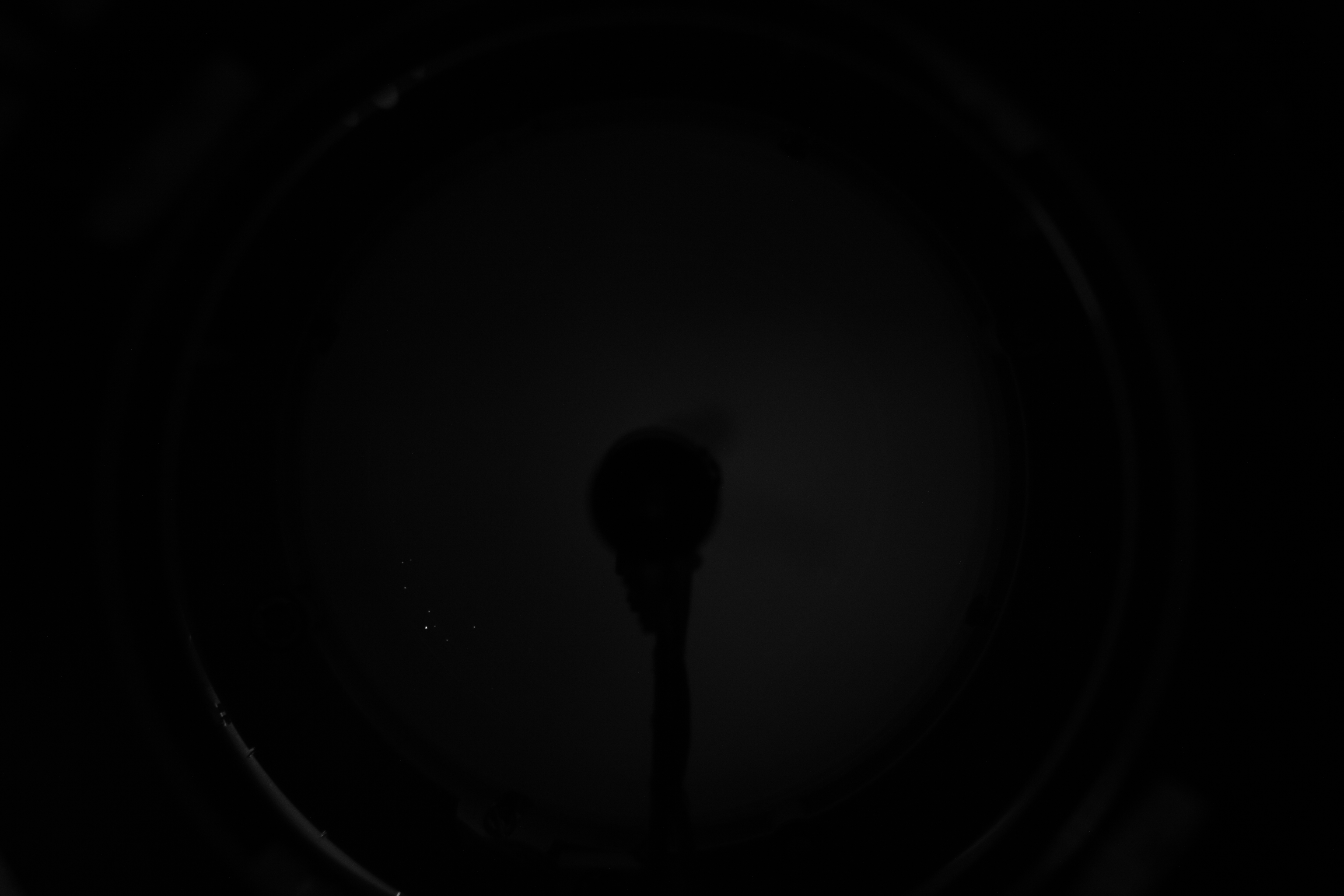

Supplement: Supplementary file 9 — Supplementary Data 7 [file 41467_2026_73690_MOESM9_ESM.zip › raw data/FigS10_LEED_rec/Fig.S10b-right_LEED_B3_after-drop/SFig.10b-right_20240425_B3_H2O_150eV_2-3A_0kV_dark.tiff]
